# MDM4 inhibits ferroptosis in p53 mutant colon cancer via regulating TRIM21/GPX4 expression

**DOI:** 10.1038/s41419-024-07227-y

**Published:** 2024-11-14

**Authors:** Jie Liu, Xujin Wei, Yixuan Xie, Yuxiang Yan, Sihui Xue, Xiangyu Wang, Han Chen, Qilong Pan, Sisi Yan, Xiaoling Zheng, Qingling Huang

**Affiliations:** 1 Department of Biochemistry and Molecular Biology, School of Basic Medical Sciences, Fujian Medical University, Fuzhou, China; 2 Department of Endoscopic Center, The Provincial Hospital, Shengli Clinical Medical College of Fujian Medical University, Fuzhou, China; 3 Endoscopic Center, The First Affiliated Hospital, Fujian Medical University, Fuzhou, China; 4 Fujian Key Laboratory of Oral Diseases, School and Hospital of Stomatology, Fujian Medical University, Fuzhou, China

**Keywords:** Ubiquitylation, Colon cancer

## Abstract

MDM4 is one of the major regulators of p53. The biological effect of MDM4 on tumor is controversial, its role and molecular mechanism in colon cancer progression and prognosis are still unclear. In this study, we identify that MDM4 is significantly overexpressed in human colon cancer and high MDM4 expression was associated with poor prognosis of colon cancer with mutant p53. MDM4 inhibits the ubiquitination of the ferroptosis marker protein GPX4 at K167 and K191 by upregulating the protein expression level of the E3 ubiquitin ligase TRIM21, which promotes the polyubiquitination of GPX4 transfer from K48- to K63- linked ubiquitination. Thereby, MDM4 enhances the stability of GPX4 protein, inhibiting ferroptosis, increasing the resistance of colon cancer patients to chemotherapy, and promoting colon cancer progression. These findings elucidate the ferroptosis inhibition effect of MDM4 via regulating TRIM21/GPX4 on p53-mutated colon cancer and provide a potential therapeutic strategy for colon cancer therapy.

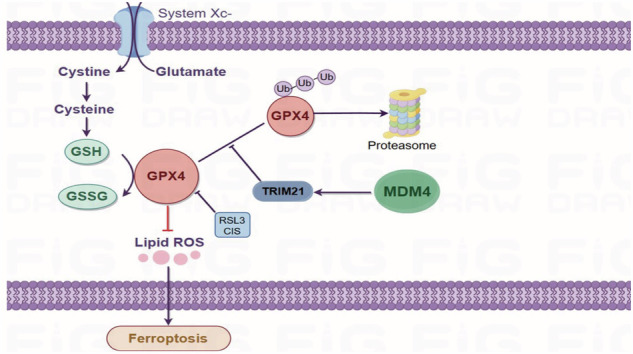

## Introduction

Colon cancer ranks among the most prevalent malignant tumors of the digestive system. A global epidemiological analysis across 185 countries revealed approximately 1.15 million new cases of colon cancer in 2020, positioning it as the fifth most common type of cancer. Concurrently, colon cancer accounted for around 580,000 deaths, making it the fifth leading cause of cancer-related mortality that year [1]. Consequently, detailed research into the molecular mechanisms driving colon cancer and the development of targeted anti-tumor therapies were crucial for improving patient survival rates.

Ferroptosis, a novel form of programmed cell death characterized by iron dependence and lipid peroxide accumulation, was first described by Dixon et al. in 2012 [2]. Emerging evidence suggested that inducing ferroptosis can inhibit colon cancer growth and overcome chemotherapy resistance [3–5]. GPX4, a member of the glutathione peroxidase family, is a selenocysteine-containing protein that reduces toxic lipid peroxides to nontoxic lipids and alcohols using glutathione, thereby preventing the formation and accumulation of lethal lipid peroxides and reactive oxygen species (ROS) [6, 7]. The human genome encodes 25 selenocysteine-containing proteins, but cells deficient in all other selenoproteins can still survive and proliferate as long as some GPX4 activity is maintained [8]. GPX4 is also essential for mouse embryonic development, with inactivation or silencing causing embryonic lethality [9]. Given its role in regulating intracellular redox homeostasis and inhibiting lipid peroxide and ROS accumulation, GPX4 emerges as a promising therapeutic target for cancer treatment.

The p53 protein, a pivotal transcription factor and tumor suppressor, plays a key role in regulating cell division, DNA damage repair, apoptosis, and senescence [10]. Mutations in p53 occur in over 50% of human tumors, with a mutation rate of 72.69% in colorectal cancer [11]. These mutations result in the loss of tumor-suppressive functions and the acquisition of gain-of-function (GOF) activities, including enhanced proliferation, survival, angiogenesis, migration, and metastasis of cancer cells [12].

MDM4, located on chromosome 1q32, encodes a protein comprising 490 amino acids. It is homologous to MDM2 and shares structural similarities, negatively regulating p53 level [13]. Recent studies indicate that MDM2 and MDM4 may also function independently of wild-type p53 [14]. Research on the p53-independent roles of MDM4 is relatively limited, with varying perspectives on its role in tumor progression. Matijasevic et al. identified that the zinc finger domain of MDM4 maintains genomic stability and suppresses tumorigenesis, proliferation, and metastasis in the presence of mutant p53 [15]. Conversely, Miranda et al. observed that MDM4 protein levels were often elevated in breast cancers expressing mutant p53, and that MDM4 knockdown inhibited the growth of mutant p53 cells both in vitro and in vivo [16]. In our study, MDM4 is found to upregulate the TRIM21 expression level to inhibit the ubiquitination of GPX4, and promotes colon cancer progression.

TRIM21, a RING-dependent E3 ubiquitin ligase, is implicated in inflammation, autoimmune diseases, and cancer progression [17]. TRIM21 exhibits dual roles in tumors, acting as both a tumor promoter and suppressor. It has been shown to promote tumor growth and proliferation by destabilizing tumor suppressor proteins such as p53 and p27 [18], and is involved in mTOR signaling [19], aerobic glycolysis [20], and amino acid metabolism [21] in cancer cells.

In this study, we demonstrate that MDM4 inhibits GPX4 degradation through TRIM21 upregulation, prevents ferroptosis, and promotes the growth of p53 mutant colon cancer cells. These findings provide a basis for understanding colon cancer progression and ferroptosis, and for identifying new targets for diagnosis and treatment.

## Materials and methods

### Cell culture and viability assay

Human cell lines HT29, SW480 and HEK293T were purchased from National Collection of Authenticated Cell Cultures (Shanghai, China) and were authenticated by STR profiling. HT29 cells were cultured in RPMI1640 medium, while SW480 and HEK293T cells were maintained in DMEM. Both media were supplemented with 10% fetal bovine serum (FBS) and 1% antibiotics. All cells were incubated at 37 °C in a humidified atmosphere containing 5% CO_2_. No mycoplasma contamination was detected.

For the viability assay, cells were seeded in 96-well plates at a density of 8000 cells per well. The following day, cells were treated with various concentrations of RSL3 (HY-100218A, MedChemExpress, the USA) or Fer-1 (HY-100579, MedChemExpress, the USA) for 48 h. Subsequently, 10% CCK8 reagent was added to each well, and cells were incubated at 37 °C for 1 h. The optical density (OD) at 450 nm was then measured to assess cell viability.

### Colony formation assay

Cells were seeded in 6-well plates at a density of 1000 cells per well and allowed to grow for 14 days. Experimental groups were treated with RSL3 at a concentration of 0.5 μM, while control groups received an equivalent concentration of DMSO. After 14 days, cells were fixed with methanol for 1 min, stained with 0.5% crystal violet at room temperature for 5 min, and carefully rinsed with tap water.

### Immunohistochemistry

Tissue sections were placed in a preheated constant-temperature incubator at 65 °C for 2 h, followed by dewaxing and hydration. Antigen retrieval was performed using the citrate buffer boiling method (0.01 M PBS, pH 6.0). After three washes with PBST, endogenous peroxidase activity was blocked by incubation at room temperature for 10 min. Sections were then incubated with the primary antibody (MDM4, A300-287A, 1:500, Bethyl Laboratories, Germany；GPX4, 67763-1-Ig, 1:1000, Proteintech, China; TRIM21, 12108-1-AP, 1:500, Proteintech, China; 4HNE, A24456, 1:100, ABclonal, China) at 4 °C overnight. The following day, a reaction enhancer was added and incubated at room temperature for 12 min, followed by the addition of a universal HRP secondary antibody and incubation for 30 min. DAB staining was performed for 1.5 min, followed by rinsing with tap water. Counterstaining with hematoxylin was conducted for 35 s, followed by bluing in ammonia water, rinsing with flowing water, dehydration, clearing, and mounting with neutral mounting medium.

The tissue microarrays used in this experiment consisted of 48 pairs of paired carcinoma and paracancerous tissues, were purchased from Shanghai Weiao Biotech Company, China.

### Quantitative real-time PCR

Total RNA was extracted using TRIzol reagent and reversely transcribed to cDNA using a Takara cDNA Synthesis Kit according to the manufacturer’s instructions. Quantitative real-time PCR was performed with SYBR Green incorporation. Relevant primer sequences were shown in Supplementary Table 1.

### Western blot

Cells were seeded in 6-well plates or 6 cm plates equally, and were lysed with RIPA lysis buffer with added protease inhibitors (P10045, 1:100 dilution; Beyotime, China). Protein concentrations were measured by the Beyotime BCA Protein Assay Kit (P0012, Beyotime, China). Protein extracts were separated by SDS-PAGE, transferred onto polyvinylidene fluoride (PVDF) membranes (P2938, Sigma-Aldrich, Germany), and blocked in 5% milk in TBST. Then the PVDF membranes were blotted individually with MDM4 (A300-287A, Bethyl Laboratories, Germany), GPX4 (67763-1-Ig, proteintech, China; 52455S, CST, America), TRIM21 (12108-1-AP, proteintech, China), Ub (10201-2-AP, proteintech, China) and β-actin (23660-1-AP, proteintech, China) antibodies. Relevant antibodies were shown in Supplementary Table 2. Three biological replicates were performed for all Western blot.

### Co-Immunoprecipitation

After lysing the cells, the cells were centrifuged at 12,000 × *g* at 4 °C for 10 min. Next, collect supernatants and incubate with different beads at 4 °C overnight on a rotating wheel. After that, wash the beads five times with Lysis buffer. Immunoprecipitates were boiled in 1× SDS loading buffer and then identify the immunoprecipitates by immunoblotting.

### Lentivirus production

After pLKO.1 vector was digested using AgeI-HF and EcoRI-HF, the corresponding shRNA was ligated to the vector, and then the plasmid was transformed and extracted. Relevant shRNA primer sequences are shown in Supplementary Table 3. All constructs were verified by sequencing. Lentiviruses were produced by co-transfection of 293T cells with 2 μg target gene plasmids construct and helper vectors (0.5 μg pMD2.G and 1.5 μg psPAX2). Viral supernatants were passed through a 0.45 um filter and concentrated with Centrifugal Filter units. Viral supernatants-infected cells were screened 48 h later using 2 μg/mL puromycin.

### ROS assay

Cells were seeded in 12-well plates at a density of 3 × 10^5^ cells per well and cultured for 14 days. The appropriate drug was added to the culture medium for 48 h. Following three washes with PBS, 2.5 μM C11 BODIPY (SML3717, Thermo Fisher, the USA) was added, and cells were incubated for 30 min at 37 °C. After incubation, the supernatant was discarded, and cells were washed three times with PBS. The cells were then digested, and the digestion was halted with complete medium before transferring into an EP tube and centrifuging at 800 rpm for 2 min. The supernatant was discarded, and the pellet was resuspended in PBS and centrifuged again. The final supernatant was discarded, and the pellet was resuspended in 500 μL of PBS before transferring to a flow-through tube for uptake and detection.

### MDA assay

Two million cells were evenly distributed in a 6-cm dish, and treatments with RSL3 and RSL3+Fer-1 were applied. After 48 h, the medium was discarded, and cells were washed three times with PBS. The MDA content was then measured following the manufacturer’s instructions (S0131, Beyotime, China).

### GSH/GSSG assay

Reagents and GSH/GSSG standard samples were prepared according to the instructions provided (S0053, Beyotime, China). After homogenizing and centrifuging the cell samples, the supernatant was mixed with the reagents and standard samples. After 25 min, absorbance was measured using an enzyme marker. The levels of GSSG and GSH were calculated based on the manufacturer’s formulas to determine the GSH/GSSG ratio.

### Ubiquitylation assay

HEK 293T cells were transfected with UB, MDM4/TRIM21, and Flag-GPX4 plasmids for 48 h, with a transfection ratio of 2:0.5:1.5. The cells were then treated with 10 μM MG132 (HY-13259, MedChemExpress, the USA) for 4 h. Post-treatment, cells were washed with PBS and lysed for an immunoprecipitation assay using 30 μL of anti-Flag beads. Western blot analysis was subsequently performed using an anti-Ub antibody to detect GPX4 ubiquitination.

### Cycloheximide chase assay

Cells were seeded into 12-well plates at a density of 300,000 cells per well. Following this, cells were treated with 2 μM cycloheximide (CHX, C7698, Sigma-Aldrich, Germany) for the indicated times and subsequently lysed for Western blot analysis.

### LC–MS/MS

Total proteins were extracted from HEK 293T cells overexpressing Flag-GPX4 using IP buffer, and precipitated with anti-Flag (HY-K0207, MedChemExpress, the USA) overnight at 4 °C. After three washes, the samples were separated by SDS-PAGE and further analyzed by mass spectrometry.

### In vivo assay

Male BALB/c nude mice, aged 4–5 weeks, were obtained from GuangDong GemPharmatech Biological Co., Ltd. The mice were housed in the Animal Experimentation Center of Fujian Medical University (Laboratory Animal License No. SCXK(Min)2012-0001) under specific pathogen-free (SPF) conditions with a normal diet. All animal experiments were approved by the Animal Ethics Committee of Fujian Medical University (Ethics No. IACUC FJMU 2024-0002).

To establish a tumor xenograft mouse model, the mice were randomly divided into four groups: Ctrl, NSC, RSL3, and NSC + RSL3. Each group contains 5 mice. Mutant p53 colon cancer cells HT29 (5 million cells in 100 μL) were injected into the right axillary skin of the mice. Tumor size and body weight were measured every two days post-inoculation. The researchers who measured tumor size and weight were not aware of the specific grouping. Once tumors reached a size of 1.5 cm³, the corresponding drugs were injected intraperitoneally. The Ctrl group received the solvent without drugs; the NSC group received the MDM4 inhibitor NSC146109 (10 mg/kg, 100 μL, HY-108638, MedChemExpress, the USA); the RSL3 group received the ferroptosis inducer RSL3 (20 mg/kg, 100 μL); and the NSC + RSL3 group received both NSC146109 (10 mg/kg, 100 μL) and RSL3 (20 mg/kg, 100 μL). The drugs were dissolved in a mixture of 10% DMSO, 40% PEG300, 5% Tween80, and 40% saline. Tumor size and body weight were measured every two days following injection. After five injections, the mice were euthanized for tumor photography, extraction, and immunohistochemical analysis.

For conditional overexpression mice, we first constructed vectors in vitro and microinjected CRISPR/Cas9 and Donor vectors into fertilized eggs of C57BL/6JGpt mice to obtain F0 generation mice. Then the F0 generation positive mice verified to be correct by PCR and sequencing were mated with C57BL/6JGpt mice to obtain the stable heritable F1 generation positive mouse model. MDM4-P2A-zsGreen gene could be expressed in colon tissues after mating with Villin-Cre tool mice. These mice and C57BL/6 wild-type (WT) mice were raised in microisolator cages with filtered air under specific pathogen-free conditions, and free access to sterile water and autoclaved food. Mice used in the experiment were aged 8-10 weeks.

### Statistical analysis

Normality of the data was assessed using normality tests. For two-group comparisons of normally distributed values that met the test for chi-square, t-tests were used, while one-way ANOVA was employed for three-group comparisons. For non-normally distributed data, Wilcoxon and Kruskal–Wallis tests were applied for two- and three-group comparisons, respectively. Data were presented in the form of mean ± SD. All statistical analyses were two-sided and considered statistically significant as *P* value < 0.05.

## Results

### MDM4 is highly expressed in colon cancer and promotes growth and proliferation of colon cancer cells harboring mutant p53

**Fig. 1:**
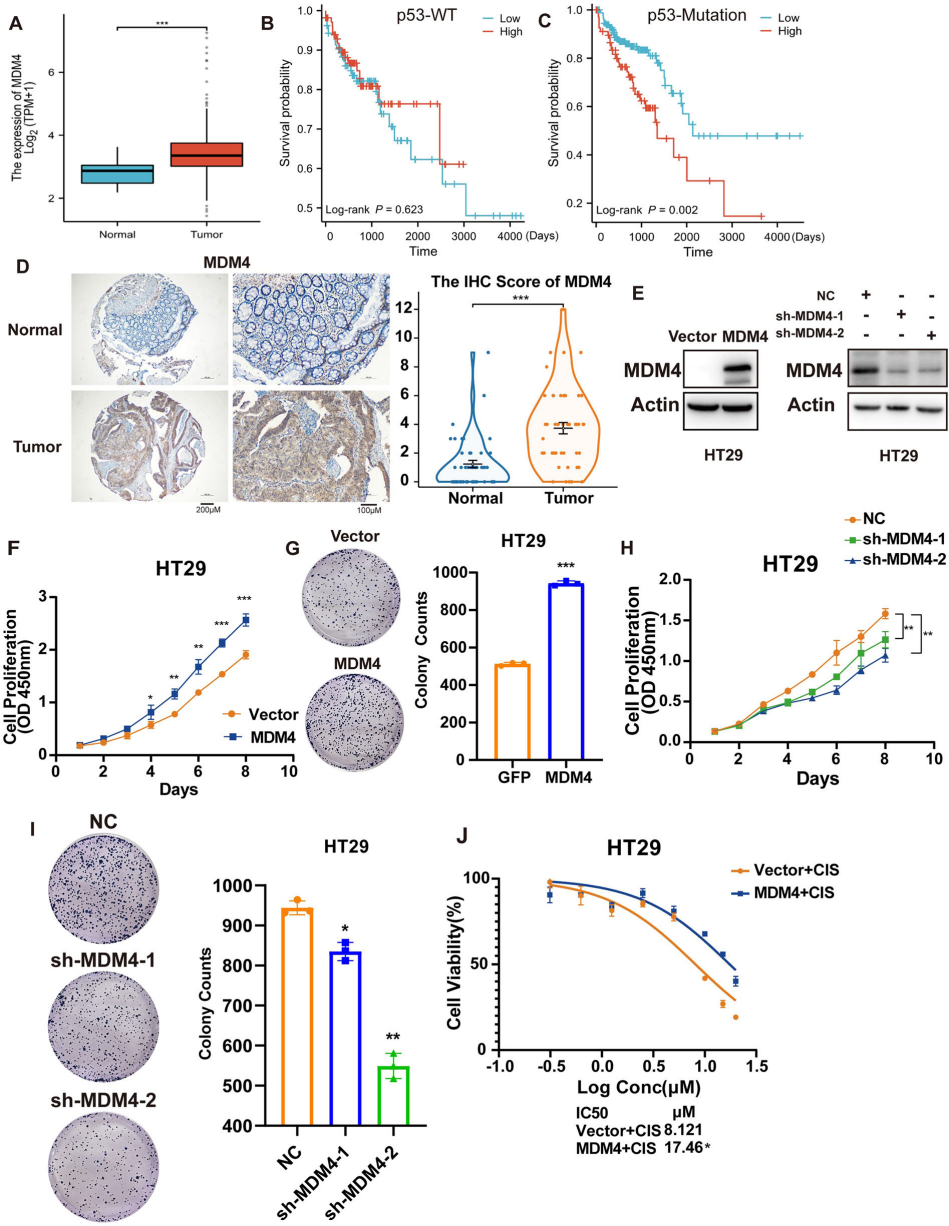
MDM4 promotes growth and proliferation of p53 mutant colon cancer cells. **A** Differential expression of MDM4 between colon cancer and normal samples based on bioinformatics analysis. **B**, **C** Kaplan–Meier survival curves comparing overall survival in patients with wild-type and mutant p53 colon cancer. **D** Immunohistochemical analysis of MDM4 expression in a tissue microarray containing 48 colon cancer specimens, with corresponding immunohistochemical scoring (*n* = 48). **E** Western blot showing MDM4 expression status in stable transfected cell lines. **F** Cell viability assessed by CCK8 assay demonstrating the effect of MDM4 overexpression on cell growth (*n* = 3). **G** Colony formation assay illustrating the effect of MDM4 overexpression on colon cancer cell proliferation (*n* = 3). **H** Cell viability assessed by CCK8 assay demonstrating the effect of MDM4 knockdown on cell growth (*n* = 3). **I** Colony formation assay illustrating the effect of MDM4 knockdown on colon cancer cell proliferation (*n* = 3). **J** Overexpression of MDM4 enhances resistance to cisplatin in HT29 cells (*n* = 3). *P < 0.05; **P < 0.01; ***P < 0.001. **D**–**G**, **J** T test and **H**, **I** One-way ANOVA were used for statistical analysis.

Our analysis of MDM4 gene expression changes in colon cancer tissues from the TCGA database, and its relationship with patient survival, revealed that MDM4 expression was significantly upregulated in colon cancer tissues compared to adjacent normal tissues (Fig. 1A). MDM4 expression had no significant effect on overall survival in p53 wild-type patients (*P* = 0.587) (Fig. 1B), but significantly affected survival in patients with mutant p53 (*P* = 0.002) (Fig. 1C). Immunohistochemical analysis of tissue microarrays containing 48 pairs of human colon cancer and adjacent normal tissues confirmed that MDM4 expression was significantly higher in colon cancer tissues, with immunohistochemical scores showing statistical significance (*P* = 0.0003) (Fig. 1D).

We selected two p53 mutant colon cancer cell lines, HT29 (R273H) and SW480 (R273H, P309S), and established stable cell lines overexpressing and knocking down MDM4 (Figs. 1E and S1A). Growth curves (Figs. 1F and S1B) and colony formation assays (Figs. 1G and S1C) indicated that overexpression of MDM4 promoted the growth and proliferation of p53 mutant colon cancer cells. Conversely, growth curves (Figs. 1H and S1D) and colony formation assays (Figs. 1I and S1E) demonstrated that knockdown of MDM4 inhibited the growth and proliferation of p53 mutant colon cancer cells. IC50 experiments showed that overexpression of MDM4 significantly decreased the sensitivity of HT29 and SW480 cells to the platinum-based chemotherapeutic agent cisplatin, increasing their IC50 from 8.12 and 10.19 µM to 17.46 and 12.95 µM, respectively, thereby greatly enhancing the resistance of colon cancer cells to cisplatin treatment (Figs. 1J and S1F).

### MDM4 inhibits ferroptosis in p53 mutant colon cancer cells

**Fig. 2:**
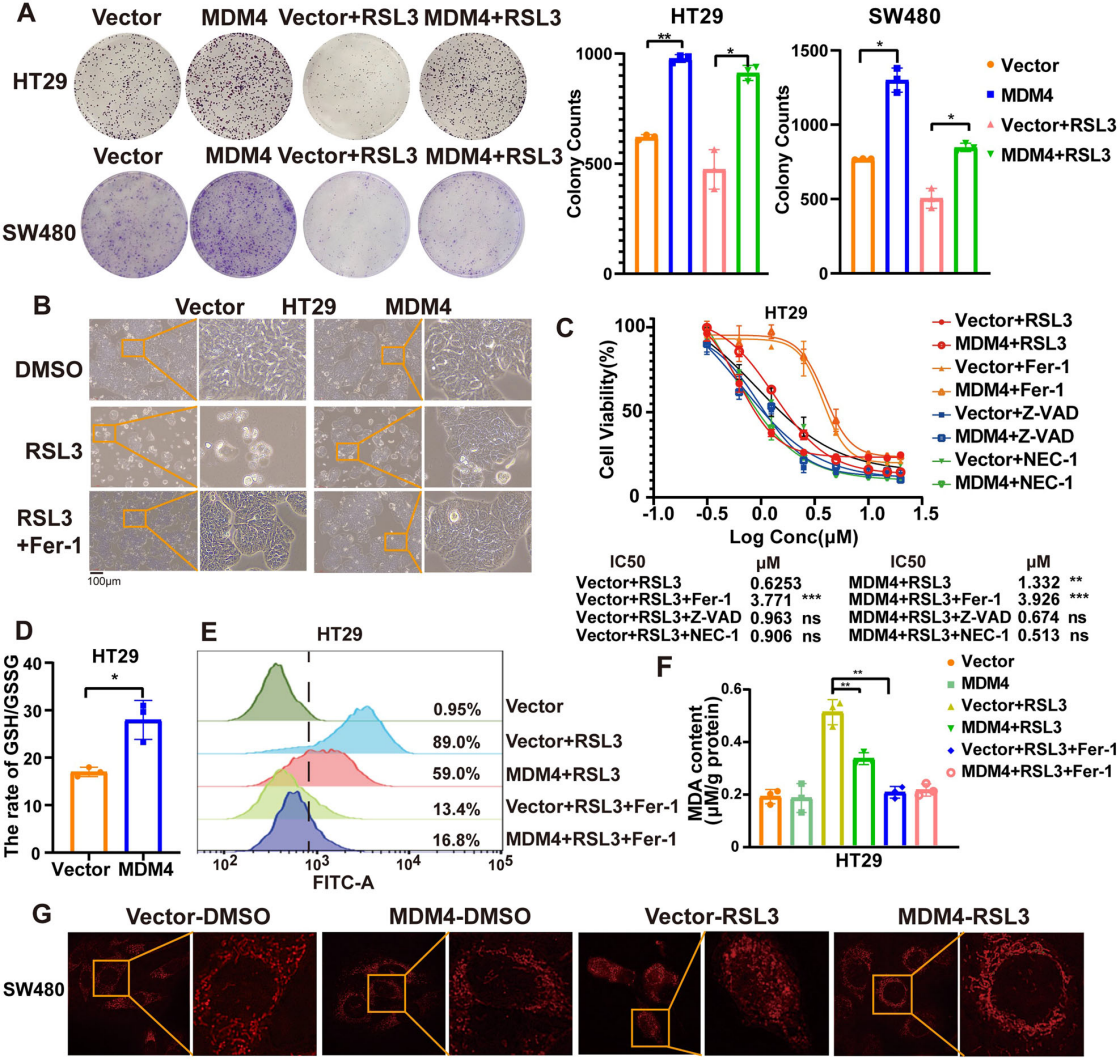
Overexpression of MDM4 inhibits ferroptosis in p53 mutant colon cancer cells. **A** Colony formation assay showed that HT29 cells overexpressing MDM4 resisted to ferroptosis induced by RSL3 (*n* = 3). **B** Microscopic images demonstrating cell morphology changes in HT29 cells after RSL3 treatment with or without MDM4 overexpression (*n* = 3). **C** Cell viability of HT29 cells treated with different doses of RSL3 alone, or in combination with Fer-1 or Z-VAD for 48 h (*n* = 3). **D** Effect of MDM4 overexpression on GSH/GSSG ratio in HT29 cells (*n* = 3). **E** Flow cytometry analysis of ROS production in MDM4 overexpression cells treated by RSL3 alone or with Fer-1. **F** MDA content measured in MDM4 overexpression cells treated by RSL3 alone or with Fer-1 (*n* = 3). **G** Ultra-high resolution microscopy images showing mitochondrial morphology in SW480 cells overexpressing MDM4 after RSL3 treatment. ns: *P* > 0.05; *P < 0.05; **P < 0.01. **A**, **C**, **F** One-way ANOVA and **D** T test were used for statistical analysis.

Cisplatin, a class II ferroptosis inducer, induces ferroptosis in colon cancer and synergistically promotes ferroptosis in colorectal cancer when combined with Erastin [22]. As previously mentioned, MDM4 enhanced the resistance of colon cancer cells to cisplatin treatment. To investigate the effect of MDM4 on ferroptosis in p53 mutant colon cancer cells, we conducted colony formation assays and cytomorphological observations. The results showed that overexpression of MDM4 promoted the proliferation of colon cancer cells and enhanced their resistance to the ferroptosis inducer RSL3 (Figs. 2A, B and S2A). Treatment with different concentrations of RSL3 revealed that overexpression of MDM4, as well as the ferroptosis inhibitor Fer-1, but not the apoptosis inhibitor Z-VAD or the necrosis inhibitor NEC-1, significantly increased cellular resistance to RSL3 compared to controls (Figs. 2C and S2B). Overexpression of MDM4 increased the GSH/GSSG ratio (Figs. 2D and S2C), resisted RSL3-induced ROS accumulation (Figs. 2E and S2D), and decreased MDA content (Figs. 2F and S2E) in p53 mutant colon cancer cells. Morphologic features of ferroptosis include small mitochondria, concentrated membrane density, decreased or vanishing mitochondrial cristae, and outer mitochondrial membrane rupture mitochondrial alterations [23]. So, we tried high-resolution microscopy to observe the mitochondrial morphology of cells. The results showed that MDM4 overexpression successfully inhibited RSL3-induced mitochondrial breakage and crumpling (Fig. 2G).

**Fig. 3:**
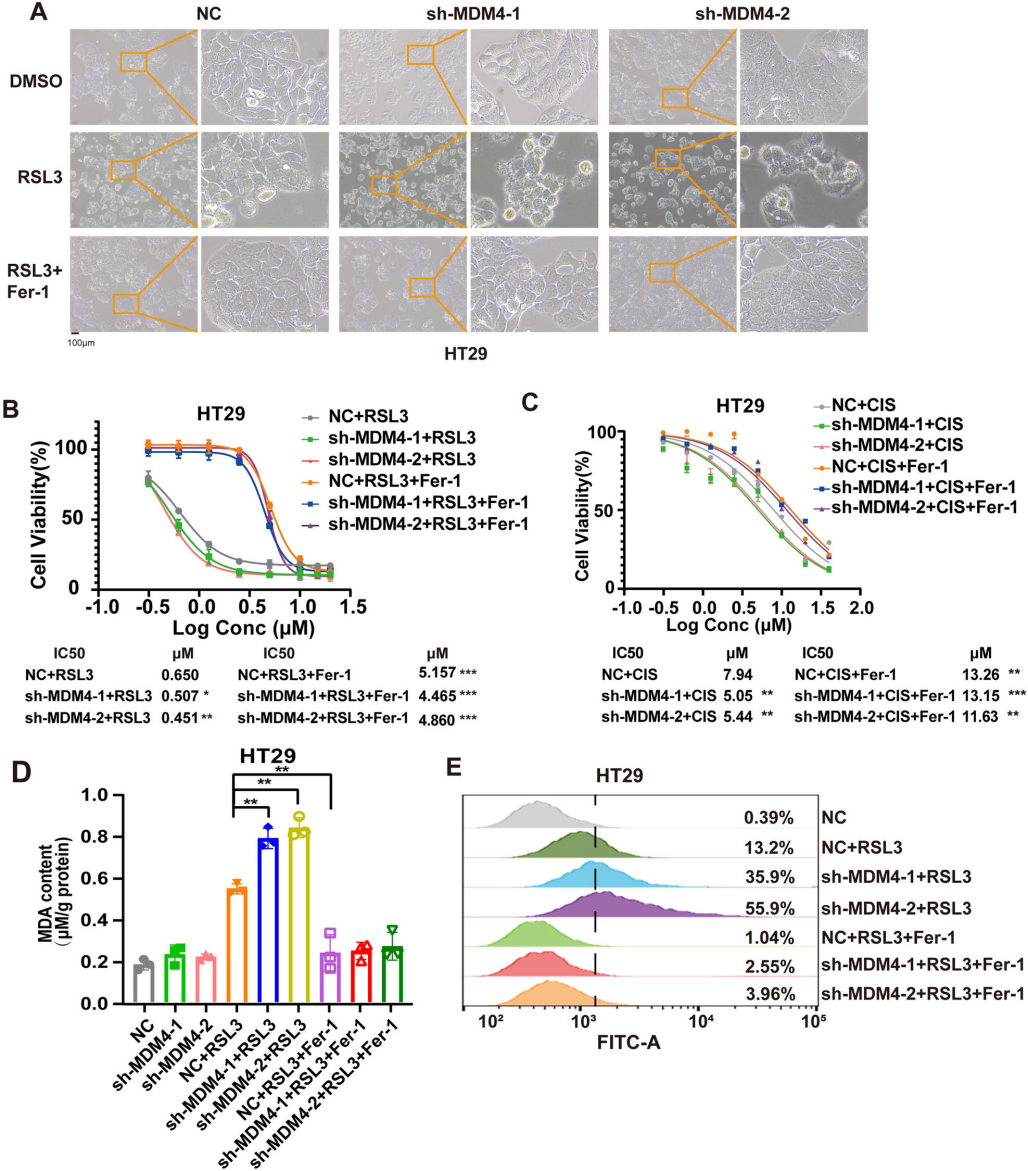
Knockdown MDM4 induces ferroptosis in p53 mutant colon cancer cells. **A** Microscopic images showing cell morphology changes in HT29 cells after RSL3 treatment with or without MDM4 knockdown. **B** Cell viability of HT29 cells treated with different doses of RSL3 alone, or in combination with Fer-1 for 48 h after MDM4 knockdown (*n* = 3). **C** Cell viability of HT29 cells treated with different doses of cisplatin (CIS) for 48 h after MDM4 knockdown (*n* = 3). **D** MDA content measured in MDM4 knockdown cells treated by RSL3 alone or with Fer-1 (*n* = 3). **E** Flow cytometry analysis of ROS production in MDM4 knockdown cells treated by RSL3 alone or with Fer-1. *P < 0.05; **P < 0.01; ***P < 0.001. **B**–**D** One-way ANOVA was used for statistical analysis.

Conversely, knockdown of MDM4 decreased the resistance of cells to RSL3 (Figs. 3A, B and S3A, B). Furthermore, MDM4 knockdown attenuated the resistance of colon cancer cells to cisplatin, and Fer-1 was able to restore the cells’ resistance to cisplatin (Figs. 3C and S3C). Similarly, knockdown of MDM4 exacerbated RSL3-induced MDA (Figs. 3D and S3D) and ROS accumulation (Fig. 3E). These experimental results indicate that MDM4 can regulate ferroptosis in colon cancer cells.

### MDM4 inhibits GPX4 degradation by switching its polyubiquitination from K48 to K63

**Fig. 4:**
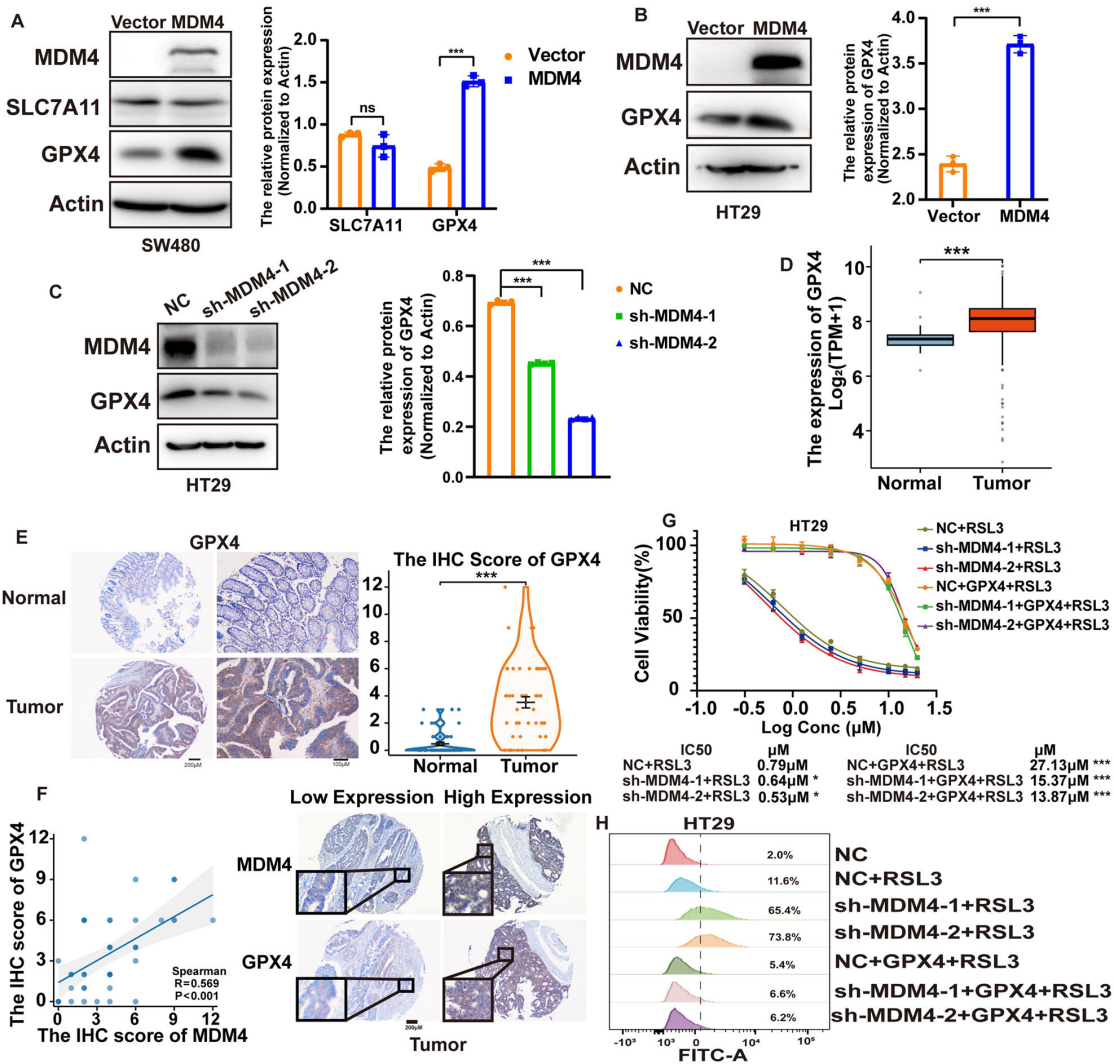
MDM4 inhibits ferroptosis by upregulating GPX4 protein expression levels. **A** Western blot showed the effect of MDM4 on the expression levels of ferroptosis-related proteins SLC7A11 and GPX4 in SW480 cells (*n* = 3). **B** Western blot demonstrated the effect of MDM4 overexpression on GPX4 protein expression levels in HT29 cells (*n* = 3). **C** Western blot showed the effect of MDM4 knockdown on GPX4 protein expression levels in HT29 cells (*n* = 4). **D** Analysis of GPX4 expression in colon cancer compared to paracancerous tissues in the TCGA database. **E** Immunohistochemical analysis of GPX4 expression level in human colon cancer tissue microarrays with paracancerous tissues (*n* = 48). **F** Correlation analysis of MDM4 and GPX4 expression using immunohistochemical scoring (*n* = 48). **G** Effect of GPX4 overexpression on RSL3 resistance in colon cancer cells following MDM4 knockdown (*n* = 3). **H** Effect of GPX4 overexpression on ROS production in mutant p53 cells following MDM4 knockdown, as detected by flow cytometry. *P < 0.05; **P < 0.01; ***P < 0.001. **A**, **B**, **E** T test, **C**, **G** One-way ANOVA and **F** Wilcoxon rank sum test were used for statistical analysis.

To explore the mechanism by which MDM4 influences ferroptosis, we conducted Western blot assays to test two most important ferroptosis-related proteins SLC7A11 and GPX4. Interestingly, MDM4 overexpression did not alter the protein expression of SLC7A11 (Fig. 4A) but significantly increased the protein levels of GPX4 (Fig. 4A, B). Conversely, knockdown of MDM4 decreased GPX4 protein expression (Figs. 4C and S4A). Analysis of the TCGA colon cancer database and subsequent immunohistochemical analysis on tissue microarrays confirmed higher GPX4 expression in colon cancer tissues compared to adjacent normal tissues (Fig. 4D, E). Correlation analysis revealed a significant positive correlation between GPX4 and MDM4 protein expression levels in these tissues (correlation coefficient R = 0.569, *P* < 0.001) (Fig. 4F).

Next, we investigated whether overexpression of GPX4 could rescue cellular resistance to RSL3 in MDM4 knockdown cells. Indeed, overexpression of GPX4 restored resistance to RSL3-induced cell death (Figs. 4G and S4B) and inhibited ROS production (Fig. 4H).

**Fig. 5:**
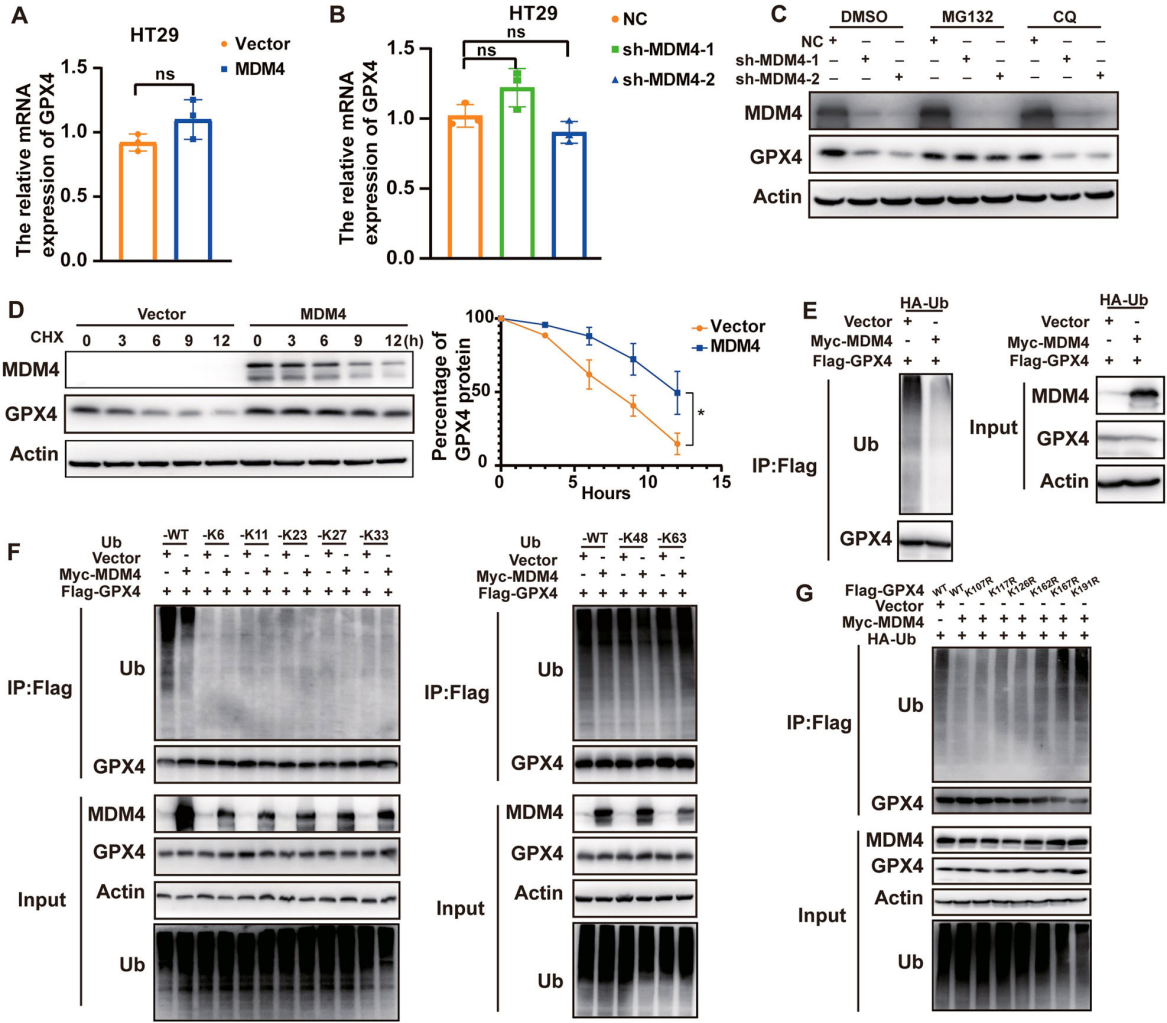
MDM4 inhibits GPX4 degradation by switching its polyubiquitination from K48 to K63. **A**, **B** Effect of MDM4 overexpression or knockdown on GPX4 mRNA levels in HT29 cells (*n* = 3). **C** Effect of MG132 and CQ treatments on changes in GPX4 protein expression following MDM4 knockdown in HT29 cells. **D** CHX treatment showed GPX4 degradation kinetics following MDM4 overexpression in HT29 cells (*n* = 3). **E** Changes of GPX4 ubiquitination level following MDM4 expression. **F** Ubiquitylation assay showed MDM4 had no significant effect on K6, K11, K27, K29, and K33 ubiquitination, and promoted GPX4 polyubiquitination shift from K48 to K63. **G** Ubiquitylation assay showed the effect of Flag-GPX4 mutants on the GPX4 ubiquitination change induced by MDM4. ns: *P*å 0.05；*P < 0.05; **P < 0.01; ***P < 0.001. **A** T test, **B** One-way ANOVA and **D** T test were used for statistical analysis.

To further elucidate how MDM4 regulates GPX4 protein levels, we examined GPX4 mRNA levels after MDM4 modulation and found no significant changes (Figs. 5A, B and S5A, B). The decrease/increase in GPX4 protein levels upon MDM4 knockdown/overexpression without a corresponding decrease/increase in mRNA suggested potential protein degradation. Treatment with the proteasome inhibitor MG132 restored GPX4 protein levels in MDM4 knockdown cells, whereas the lysosomal inhibitor chloroquine did not (Fig. 5C). Moreover, overexpression of MDM4 protected GPX4 from degradation induced by the protein synthesis inhibitor CHX (Fig. 5D), suggesting a role for MDM4 in stabilizing GPX4 protein levels. Given that protein stability is primarily regulated by the ubiquitination system, we investigated whether MDM4 affects GPX4 ubiquitination. Co-immunoprecipitation assays in HEK293T cells overexpressing HA-Ub, Flag-GPX4, and Myc-MDM4 demonstrated that MDM4 overexpression inhibited the ubiquitination and subsequent degradation of GPX4 (Fig. 5E). To determine the specific type of ubiquitination involved, we generated ubiquitin mutants with single lysine residues preserved: HA-Ub K6 (where other lysine residues were mutated to arginine except K6), HA-Ub K11, HA-Ub K23, HA-Ub K27, HA-Ub K33, HA-Ub K48, and HA-Ub K63. These constructs were co-expressed with Flag-GPX4 and Myc-MDM4 in HEK293T cells, followed by Co-immunoprecipitation (Co-IP) assays. Our findings indicate that MDM4 overexpression significantly enhances K63-linked polyubiquitination of GPX4 while concurrently reducing K48-linked polyubiquitination (Fig. 5F). K48-linked ubiquitin chains normally degrade proteins via the ubiquitin-proteasome pathway [24]. K63-linked linear or branched/mixed chains can form scaffolding platforms that play non-degrading roles in further protein stability, cellular localization and protein-protein interactions [25]. Consequently, this result suggested that MDM4 inhibited GPX4 degradation and improved the protein stability of GPX4. To further determine the ubiquitination site(s) of GPX4, we used an online site (https://www.phosphosite.org/) to predict it. We mutagenized every Lys to Arg and co-transfected each GPX4 mutant with HA-Ub and MDM4 into HEK293T cells. Two mutations (K167R and K191R) displayed a reduction in MDM4-mediated ubiquitination change (Fig. 5G).

### MDM4 inhibits GPX4 ubiquitination and upregulates its protein level through TRIM21

**Fig. 6:**
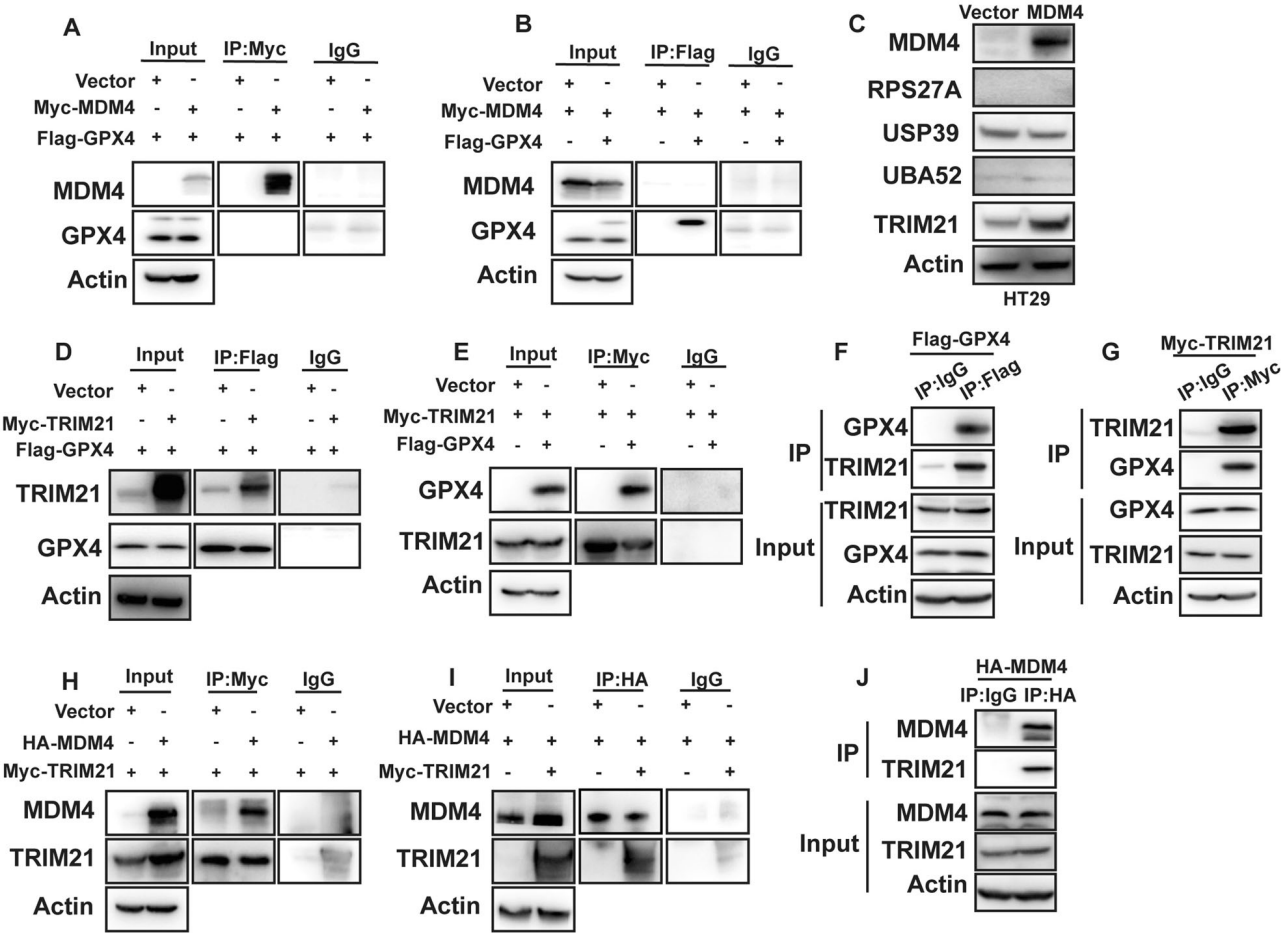
MDM4 upregulates GPX4 through TRIM21. **A** Immunoprecipitation of Myc-tagged MDM4 detected interaction with GPX4. **B** Immunoprecipitation of Flag-tagged GPX4 detected interaction with MDM4. **C** Effect of MDM4 overexpression on the expression level of the GPX4-interacting E3 ubiquitin ligase identified by mass spectrometry assay in HT29 cells. Immunoprecipitation of **D** Flag-tagged GPX4 detects interaction with Myc-TRIM21, **E** Myc-tagged TRIM21 detects interaction with Flag-GPX4, **F** Flag-tagged GPX4 detects interaction with endogenous TRIM21, **G** Myc-tagged TRIM21 detects interaction with endogenous GPX4, **H** Myc-tagged TRIM21 detects interaction with HA-MDM4, **I** HA-tagged MDM4 detects interaction with Myc-TRIM21, **J** HA-tagged MDM4 detects interaction with endogenous TRIM21.

Our investigation into the mechanism by which MDM4 influences GPX4 protein levels revealed that MDM4 does not directly interact with GPX4 (Fig. 6A, B). To explore this further, we conducted immunoprecipitation of GPX4 followed by mass spectrometry analysis (Fig. S5C). Through literature review and data analysis, we identified several E3 ubiquitin ligases potentially modulated by MDM4. Notably, we found that MDM4 overexpression upregulates TRIM21 protein expression (Figs. 6C and S6A). Then we overexpressed Myc-TRIM21 and Flag-GPX4 in HEK293T cells and conducted Co-IP assays (Fig. 6D, E), and we overexpressed Flag-GPX4 in HEK293T cells and Myc-TRIM21 in HT29 cells to confirm that overexpressed Flag-GPX4 and Myc-TRIM21 could bind to endogenous TRIM21 and GPX4 separately (Fig. 6F, G). We further overexpressed Myc-TRIM21 and HA-MDM4 in HEK293T cells and conducted Co-IP assays (Fig. 6H, I), and we overexpressed HA-MDM4 in HT29 cells and conducted Co-IP assays (Fig. 6J). The results confirmed the binding between TRIM21 and MDM4.

**Fig. 7:**
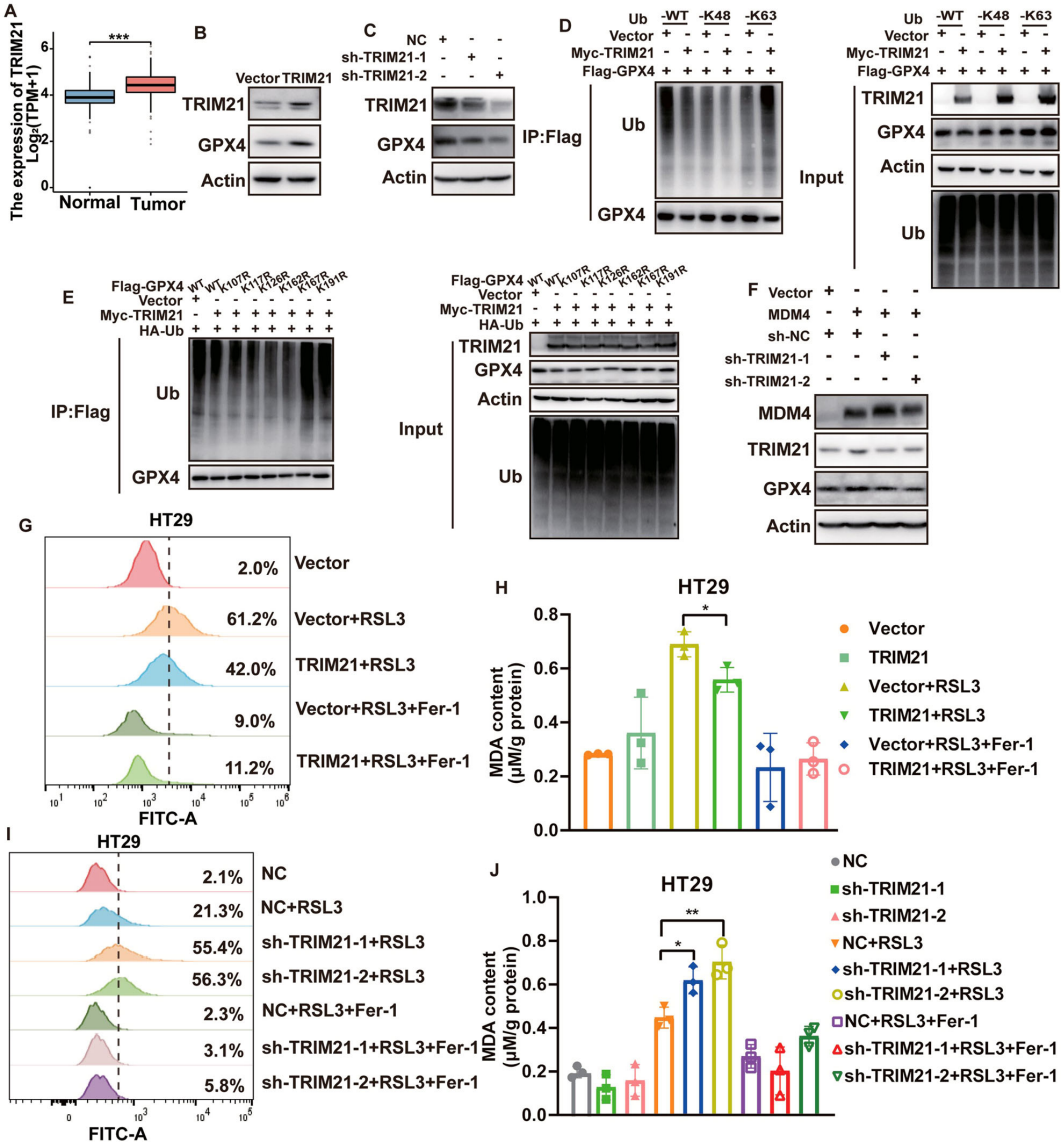
MDM4 upregulates GPX4 protein expression levels through TRIM21. **A** Expression levels of TRIM21 in colon cancer compared to normal tissues from TCGA and GTEx databases. **B** Western blot showed the effect of TRIM21 overexpression on GPX4 protein expression levels in HT29 cells. **C** Western blot showed the effect of TRIM21 knockdown on GPX4 protein expression levels in HT29 cells. **D** Ubiquitylation assay showed that TRIM21 promoted the polyubiquitination transition of GPX4 from K48 to K63. **E** Ubiquitylation assay showed the effect of Flag-GPX4 mutants on the GPX4 ubiquitination change induced by TRIM21. **F** Western blot showed the change in GPX4 expression after TRIM21 knockdown in HT29 cells overexpressing MDM4. **G** Flow cytometry analysis of ROS production in TRIM21 overexpressing cells. **H** MDA content measured in TRIM21 overexpressing cells (*n* = 3). **I** Flow cytometry analysis of ROS production in TRIM21 knockdown cells. **J** MDA content measured in TRIM21 knockdown cells (*n* = 3). ns: *P >* 0.05；*P < 0.05; **P < 0.01; ***P < 0.001. **H**, **J** One-way ANOVA were used for statistical analysis.

Analyzing RNA sequencing data from TCGA and GTEx databases revealed elevated TRIM21 expression in colon cancer tissues compared to normal tissues (Fig. 7A). To further investigate, we established cell lines with stable TRIM21 overexpression and knockdown. Western blot analysis confirmed that TRIM21 positively regulates GPX4 protein expression (Fig. 7B, C). Similar to MDM4, TRIM21 was found to enhance K63-linked polyubiquitination of GPX4 and decrease K48-linked polyubiquitination (Fig. 7D) and two GPX4 mutations (K167R and K191R) displayed a reduction in TRIM21-mediated ubiquitination change (Fig. 7E). Furthermore, knockdown of TRIM21 in HT29 cells overexpressing MDM4 attenuated the MDM4-induced upregulation of GPX4 expression (Fig. 7F).

Our experiments showed that TRIM21 overexpression in HT29 cells increased resistance to RSL3, as evidenced by a higher IC50 (Fig. S6B). Overexpression of TRIM21 also reduced RSL3-induced ROS accumulation (Fig. 7G) and MDA content (Fig. 7H). Conversely, knockdown of TRIM21 in HT29 cells sensitized cells to RSL3-induced effects (Figs. S6C and 7I, J). These implicated TRIM21 as a negative regulator of ferroptosis. These findings underscore the role of MDM4 in modulating GPX4 levels via TRIM21-mediated ubiquitination, thereby impacting ferroptosis sensitivity in p53 mutant colon cancer cells and potentially influencing therapeutic responses.

### MDM4 inhibitors work together with ferroptosis inducers to inhibit p53 mutant colon cancers growth in vivo

**Fig. 8:**
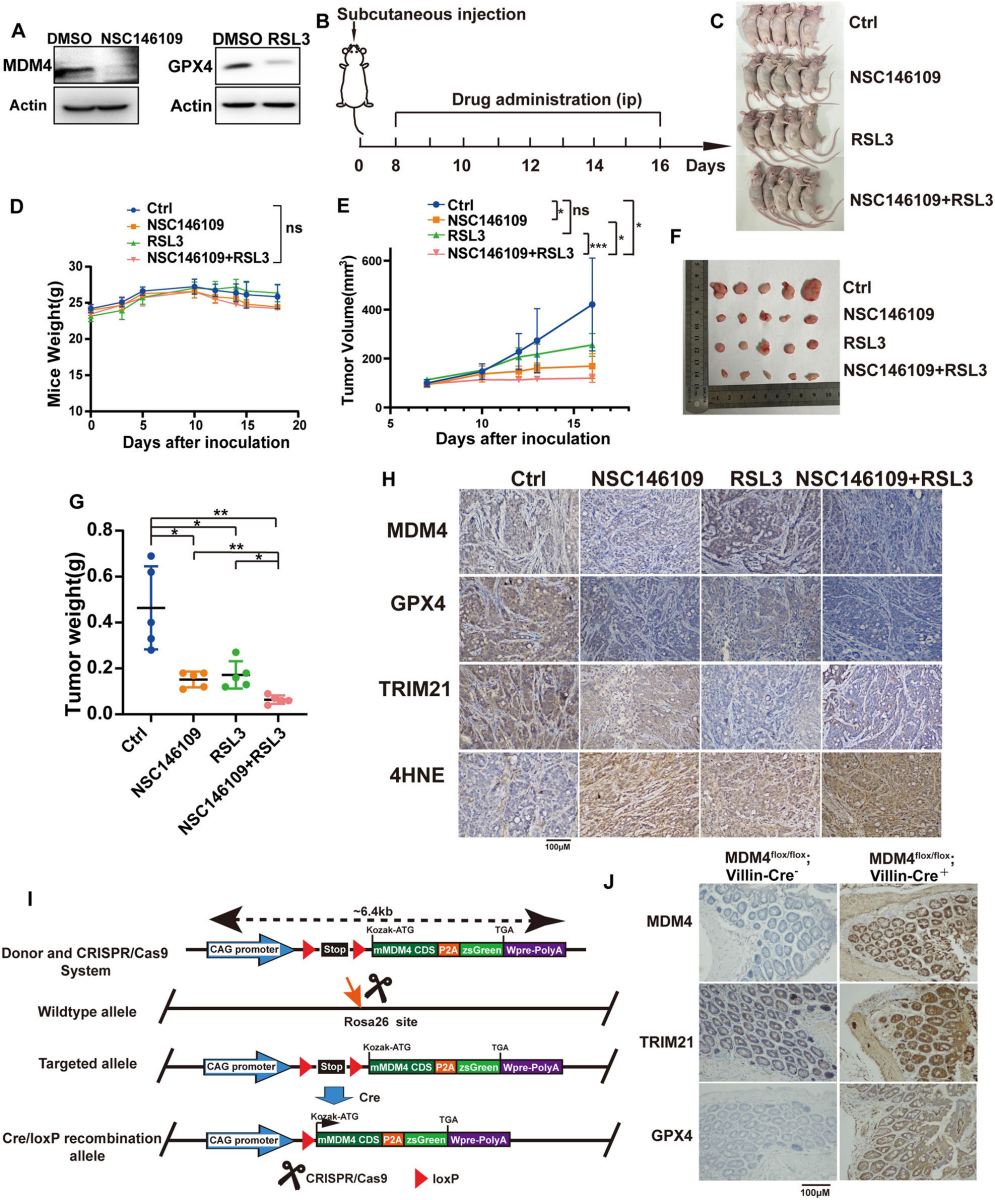
Mouse experiments demonstrate that inhibition of MDM4 enhances the sensitivity of colon cancer graft tumors to RSL3. **A** Western blot showed the effect of NSC146109 on MDM4 protein expression and RSL3 on GPX4 protein expression. **B** Schematic diagram of the intraperitoneal drug administration process in nude mice. **C** Diagram illustrating tumor formation in nude mice injected with HT29 cells after drug treatment. **D** Changes in body weight of nude mice in each treatment group. **E** Subcutaneous tumor sizes at different time points after inoculation in nude mice. **F** Graph showed subcutaneous tumors collected from nude mice after necropsy. **G** Weights of subcutaneous tumors collected from nude mice after necropsy. **H** Immunohistochemical analysis of protein expression in subcutaneous tumors from nude mice. **I** Flowchart of tissue-specific expressing MDM4 mouse construction. **J** Expression levels of TRIM21 and GPX4 in colon tissues from tissue-specific expressing MDM4 mice. ns: *P* ≥ 0.05; *P < 0.05; **P < 0.01; ***P < 0.001.

To comprehensively assess the impact of MDM4 inhibition in colon cancer, we established in vivo xenograft models using MDM4-overexpressing HT29 cells in BALB/c nude mice. We investigated the therapeutic effects of the MDM4 inhibitor NSC146109 and the ferroptosis inducer RSL3 through intraperitoneal injections. Prior to cell inoculation, we confirmed the efficacy of NSC146109 in reducing MDM4 protein levels and observed that RSL3 significantly decreased GPX4 expression at the cellular level (Fig. 8A). Mice were randomly divided into four groups: solvent control, NSC146109 alone, RSL3 alone, and combination therapy with NSC146109 and RSL3.

Tumors were initiated by injecting p53 mutant HT29 cells into the mice’s right axillary skin, reaching approximately 0.3 × 0.3 cm in size after 8 days of tumor growth and drug administration was initiated. After 16 days of treatment, mice were euthanized, and tumor samples were collected (Fig. 8B, C). Throughout the study, we monitored the mice’s body weights, observing no significant differences among the four groups (Fig. 8D). Subcutaneous tumor volumes were measured, demonstrating that both NSC146109 and RSL3 individually inhibited tumor growth, with the most pronounced effect observed in the combination therapy group (Fig. 8E, F). Consistently, both NSC146109 and RSL3 significantly reduced tumor weights, with the combination therapy group showing the most substantial reduction (Fig. 8G).

Immunohistochemical analysis of tumor samples from the four groups revealed several key findings (Fig. 8H). NSC146109 effectively reduced MDM4 expression levels, while RSL3 treatment significantly decreased GPX4 expression. Importantly, the combination of NSC146109 and RSL3 showed enhanced effects on reducing GPX4 levels and increasing 4HNE expression, a marker of lipid peroxidation associated with ferroptosis. The expression levels of TRIM21 followed a pattern similar to GPX4, indicating a potential regulatory link in vivo.

In addition, we constructed transgenic mice overexpressing MDM4 by CRISPR/Cas9 technology and crossed them with Villin-Cre mice (Fig. 8I), and genotyped them to obtain colonic conditional overexpression of MDM4 (Fig. S6D). Then we performed immunohistochemical analysis of their colon tissues, which showed elevated protein levels of TRIM21 and GPX4 (Fig. 8J).

In summary, our findings confirm that MDM4 inhibition enhances the sensitivity of p53 mutant colon cancer cells to ferroptosis induction by stabilizing GPX4 through TRIM21-mediated mechanisms. This study underscores the therapeutic potential of targeting MDM4 in combination with ferroptosis inducers for treating colon cancers harboring p53 mutations.

## Discussion

Colon cancer represents a significant health challenge globally, ranking as the fifth leading cause of cancer-related deaths. Despite advancements in clinical management, including surgery and radiotherapy, high recurrence rates persist, underscoring the need for targeted therapies and deeper molecular insights into disease mechanisms to improve patient outcomes [26]. Therefore, in-depth research on the molecular mechanism of colon cancer development and its related influencing factors, and further development of corresponding anti-tumor therapy of targeted drugs, can effectively improve the survival rate of colon cancer patients.

The role of MDM4 in cancer remains controversial in current literature. Studies have shown diverse effects of MDM4 depending on context and cancer type. For instance, MDM4 inhibitor NSC146109 has demonstrated potent anti-proliferative effects in cervical cancer by stabilizing p53 and enhancing its transcriptional activity, thereby promoting apoptosis and sensitizing cells to chemotherapy [27]. Conversely, other studies highlight MDM4’s role in maintaining genome stability and suppressing metastatic potential [15]. In our study, bioinformatics analysis and immunohistochemistry of clinical samples revealed that MDM4 expression was markedly elevated in colon cancer tissues compared to adjacent normal tissues. Importantly, high MDM4 expression correlated with poorer prognosis in patients with p53-mutant colon cancer. Overexpression of MDM4 inhibited ferroptosis in mutant p53 colon cancer, thereby promoting the development of mutant p53 colon cancer.

GPX4 was shown to be a key upstream regulator of ferroptosis in 2014 [28, 29]. It works by reducing complex hydroperoxides, including phospholipid hydroperoxides and cholesterol hydroperoxides, to their corresponding counterparts, thereby interrupting lipid peroxidation and inhibiting ferroptosis. In this study, the results showed that overexpression of MDM4 in mutant p53 colon cancer cells was able to increase the expression level of GPX4, and knockdown of MDM4 was followed by a decrease in the protein expression level of GPX4, which also provided strong evidence for the conclusion that MDM4 inhibited ferroptosis in mutant p53 colon cancer. Copper chelators promoted ubiquitinated degradation of GPX4 by directly binding to GPX4 protein cysteines C107 and C148, thereby promoting ferroptosis [30]. Yue Wang et al. found that activation of Wnt/β-catenin protein signaling attenuated ROS production, and the β-catenin/TCF4 transcriptional complex directly bound to the promoter region of GPX4 and induced its expression, thereby inhibiting ferroptosis in gastric cancer cells [31]. These studies have demonstrated the critical role of GPX4 as an important upstream regulator of ferroptosis. In this study, we found that overexpression of MDM4 in mutant p53 colon cancer cells was able to increase the expression level of GPX4, and knockdown of MDM4 was followed by a decrease in the protein expression level of GPX4, which also provided strong evidence for the conclusion that MDM4 inhibited ferroptosis in mutant p53 colon cancer.

In recent years, GPX4 has become a research hotspot in ferroptosis. Proteasomal degradation is one of the major degradation modes of GPX4 [32], and the E3 ligase associated with GPX4 proteasomal degradation has not yet been identified. Wang Z et al. showed that the E3 ubiquitin ligase TRIM26 directly interacts with GPX4 through its cyclic domain and catalyzes the ubiquitination of GPX4 at the K107 and K117 sites, which promotes the polyubiquitination transition of GPX4 from K48 to K63 and thus enhances the stability of the GPX4 protein [33]. GPX4 resists ferroptosis by recruiting LUBAC to catalyze the formation of its own linear ubiquitin chain [32]. In the present study, we observed that MDM4 inhibits GPX4 ubiquitination degradation thereby resisting ferroptosis.

TRIM21 was one of the E3 ubiquitin ligase which involved in tumor development [20]. It exhibited dual tumor-suppressive and oncogenic functions in tumors. TRIM21 mediates ubiquitination of the Hippo pathway kinase MST2 and reduces colorectal cancer metastasis [34]. TRIM21 was unable to interact with wild-type p53, but could interact directly with mutant p53, leading to ubiquitination and degradation of mutant p53, thereby inhibiting the role of mutant p53 “gain of function” in tumorigenesis [35]. These experimental results reveal the important tumor suppressor function of TRIM21. Wang Fang et al. concluded that TRIM21 expression was positively correlated with hepatocellular carcinoma (HCC), and TRIM21 knockout mice resisted hepatocellular carcinoma induced by the hepatocarcinogenic drug DEN [36]. TRIM21 promoted degradation of mitochondrial voltage-dependent anion-selective channel protein 2 (VDAC2) through K48-linked ubiquitination in nasopharyngeal carcinoma, thereby inhibiting type I interferon response after radiotherapy in nasopharyngeal carcinoma [37]. In the field of ferroptosis research, Jun Gong et al. showed that TRIM21 resisted ferroptosis by mediating K63 ubiquitination of FSP1 at the K322 and K366 residues [38]. The results of this study indicate that TRIM21 inhibits GPX4 ubiquitination degradation, increases GPX4 protein stability, inhibits ferroptosis, and promotes the development of mutant p53 colon cancer.

Our study represents the first comprehensive exploration of MDM4’s regulation of ferroptosis in colon cancer independently of wild-type p53, specifically in the context of p53 mutations. However, certain aspects remain unexplored, such as the specific structural domains of MDM4 involved in regulating GPX4 and the precise mechanisms through which MDM4 modulates TRIM21 activity. Future research will focus on elucidating these mechanisms to further refine our understanding and potentially identify novel therapeutic targets for colon cancer.

In conclusion, our findings highlight MDM4 as a critical regulator of ferroptosis in p53-mutant colon cancer, offering insights into potential therapeutic strategies targeting MDM4 and ferroptosis pathways to improve outcomes for patients with this challenging disease.

## Supplementary information

Full and uncropped Western Blot

Supplementary material

## Data Availability

The data generated in this study are available upon request from the corresponding author.
