# Supplementary material for: MDM4 inhibits ferroptosis in p53 mutant colon cancer via regulating TRIM21/GPX4 expression
Source: Cell Death Dis. 2024 Nov 14;15(11):825. doi: 10.1038/s41419-024-07227-y (PMC11564821; doi:10.1038/s41419-024-07227-y)
Supplement: Supplementary file 1 — Full and uncropped Western Blot [file 41419_2024_7227_MOESM1_ESM.docx]

Fig. 1E


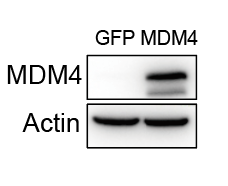


Fig. 1E MDM4


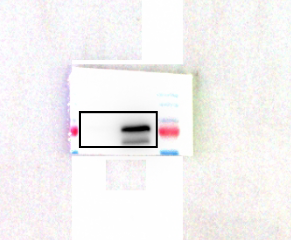


Fig. 1E Actin


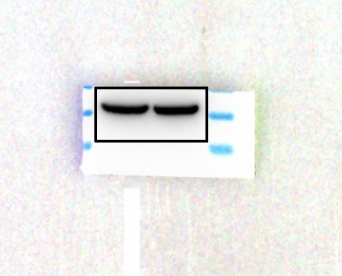


Fig. 1E


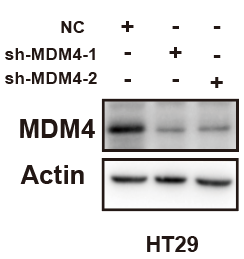


Fig. 1E MDM4


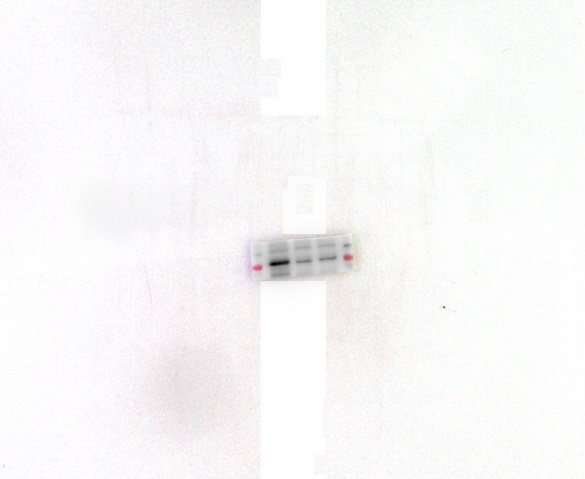


Fig. 1E Actin


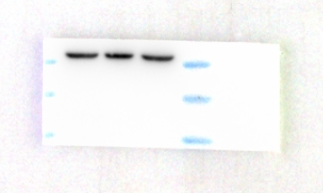


Fig. 4A


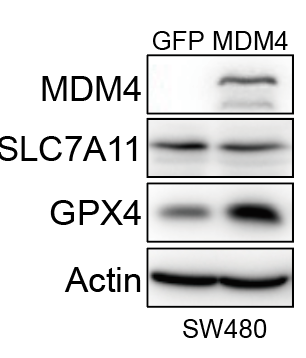


Fig. 4A Actin


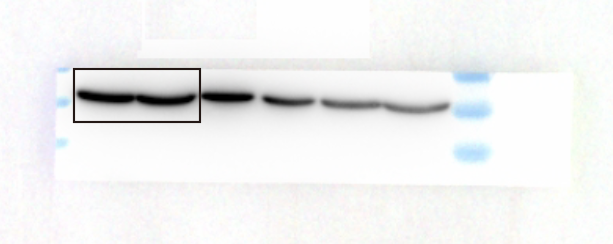


Fig. 4A GPX4


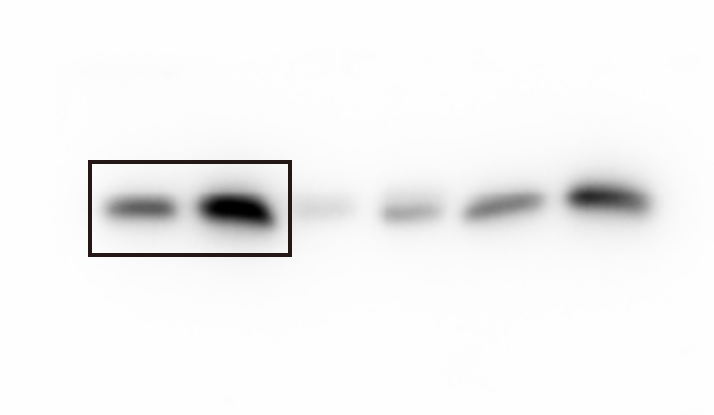


Fig. 4A MDM4


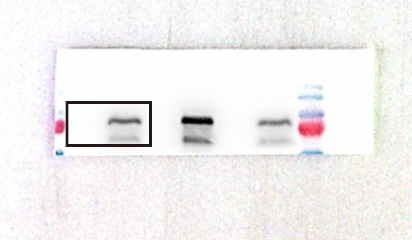


Fig. 4A SLC7A11


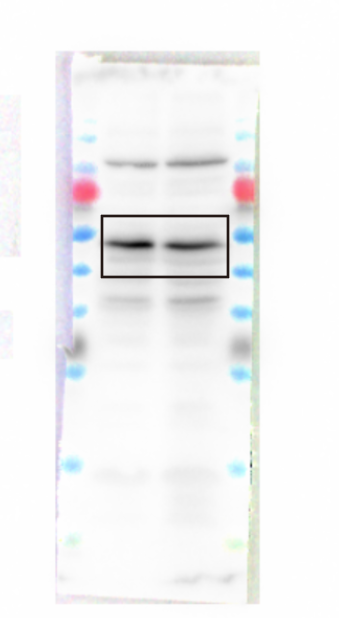


Fig. 4B


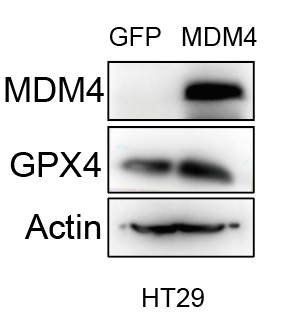


Fig. 4B Actin


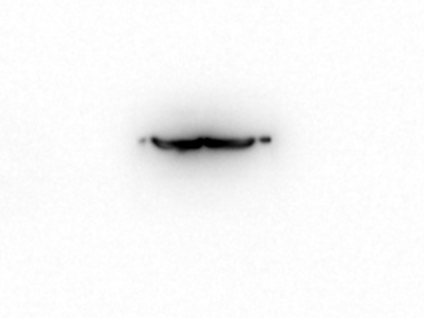


Fig. 4B GPX4


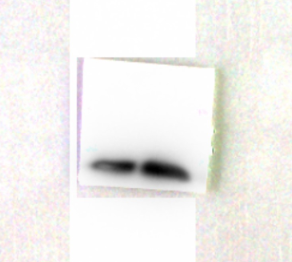


Fig. 4B MDM4


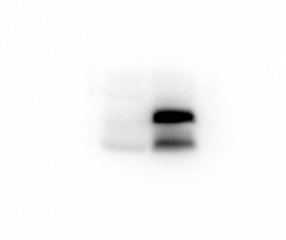


Fig. 4C


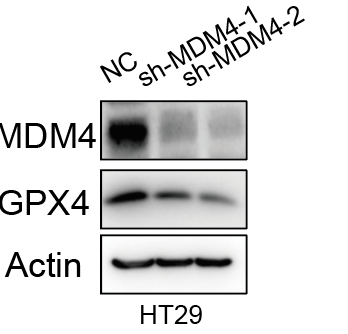


Fig. 4C Actin


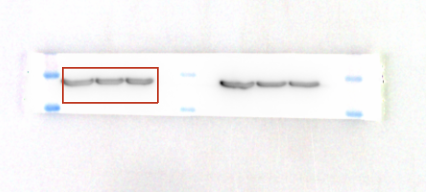


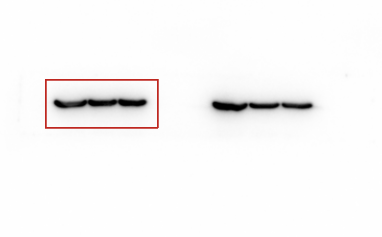


Fig. 4C MDM4


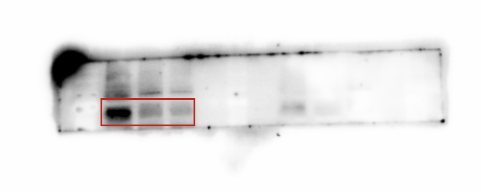


Fig. 4C GPX4


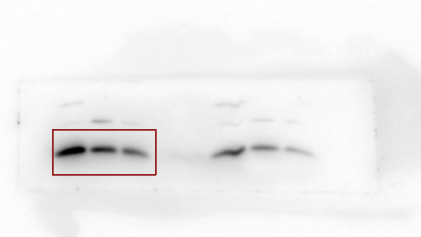


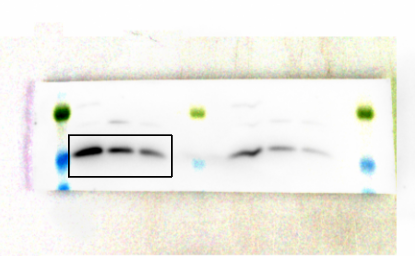


Fig. 5C


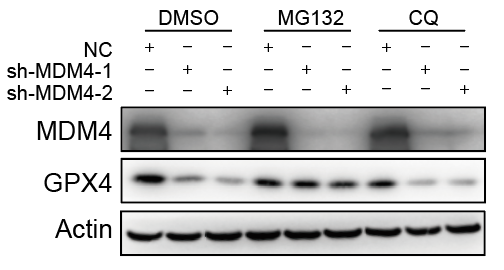


Fig. 5C Actin


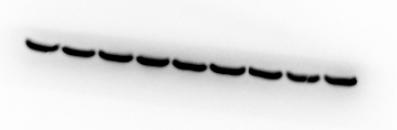


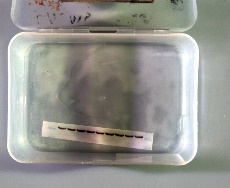


Fig. 5C GPX4


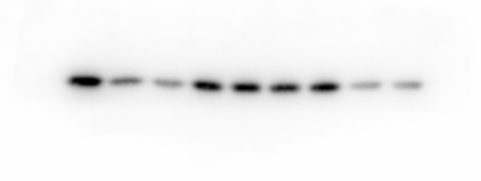


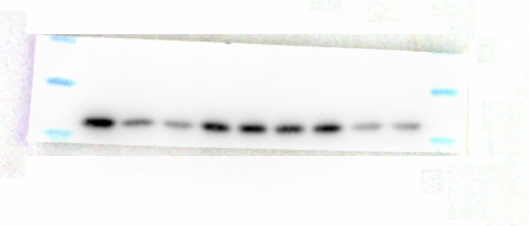


Fig. 5C MDM4


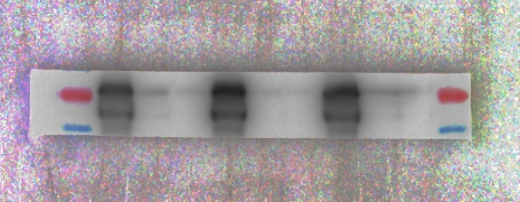


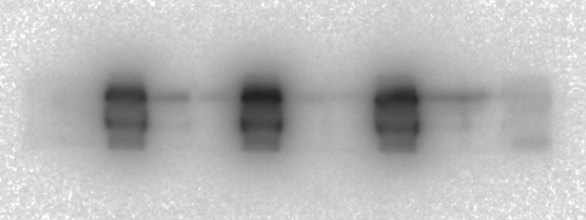


Fig. 5D


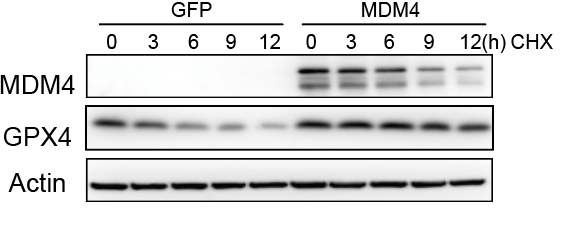


Fig. 5D MDM4


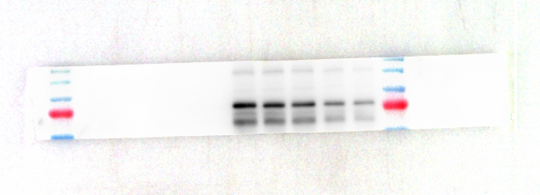


Fig. 5D GPX4


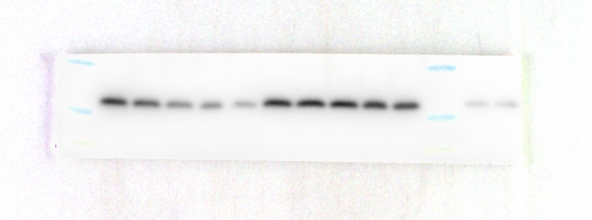


Fig. 5D Actin


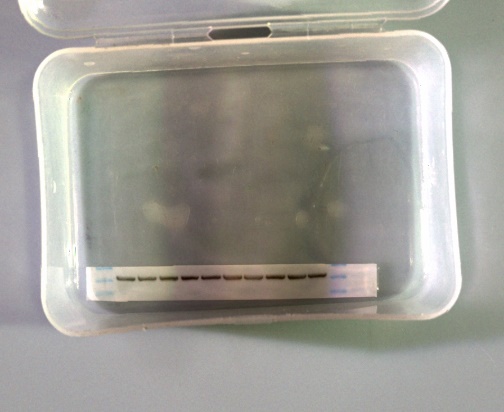


Fig. 5E


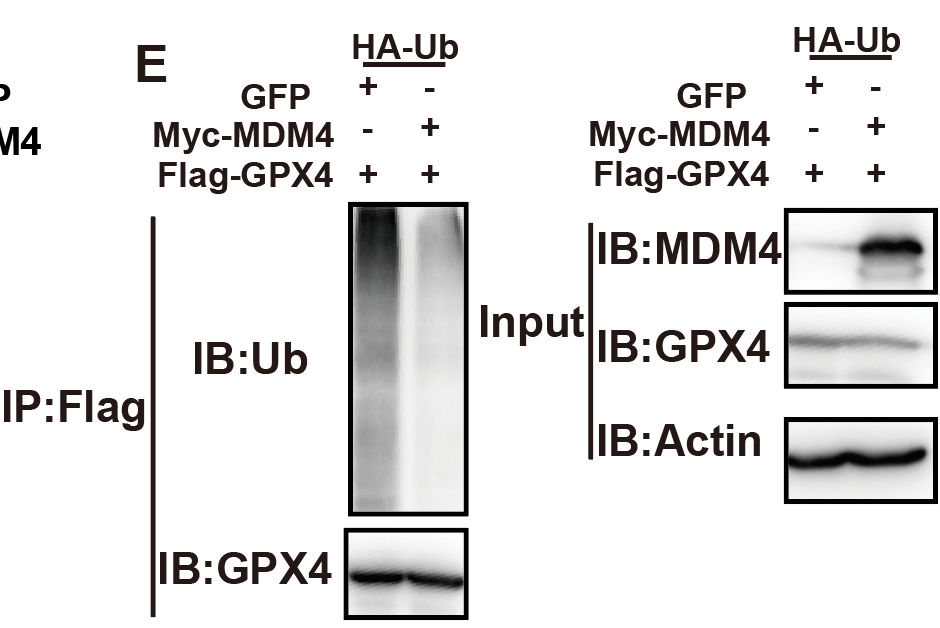


Fig. 5E Ub


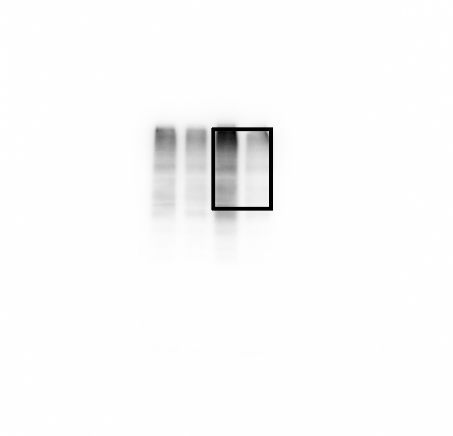


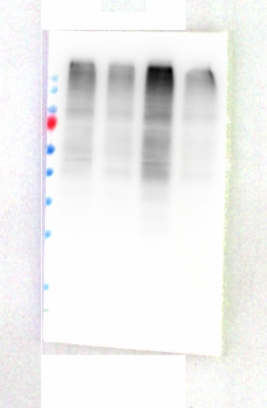


Fig. 5E GPX4


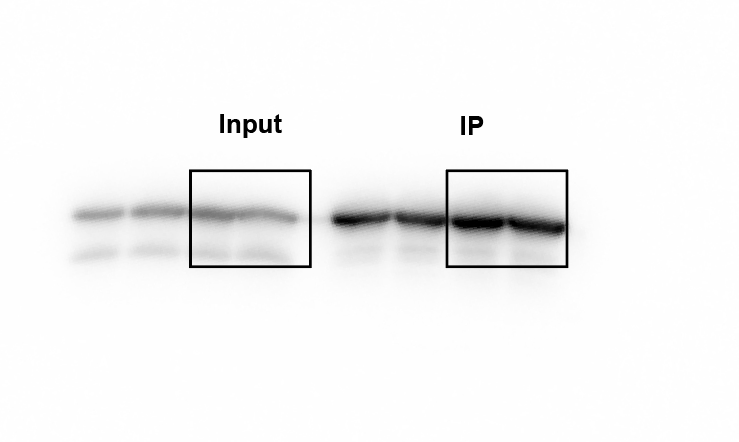


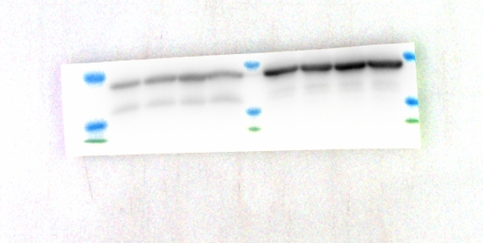


Fig. 5E MDM4


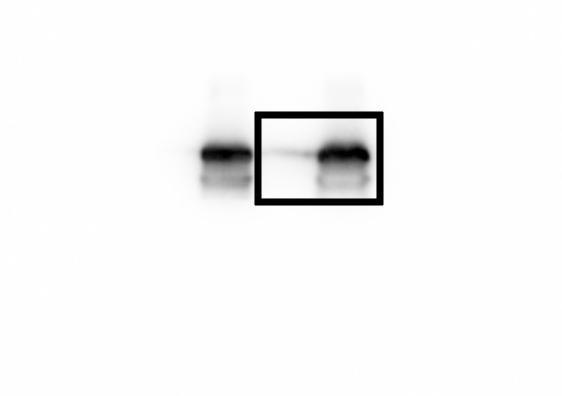


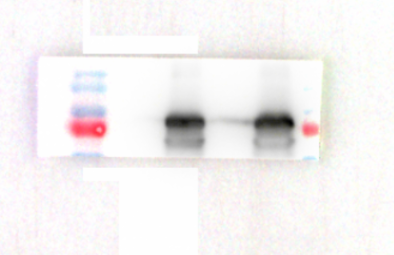


Fig.5E Actin


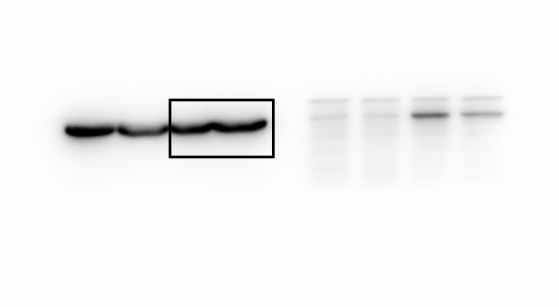


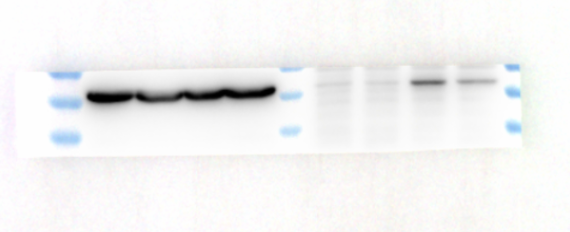


Fig. 5F


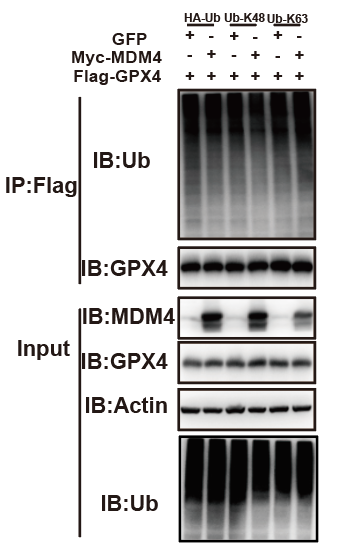


Fig. 5F Ub


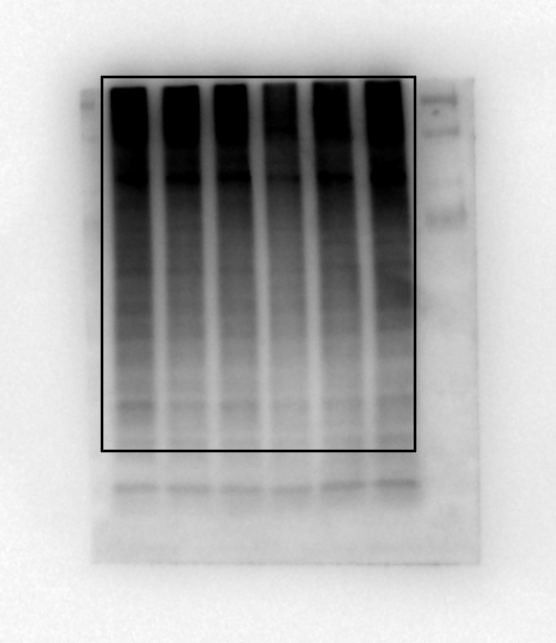


Fig. 5F Actin


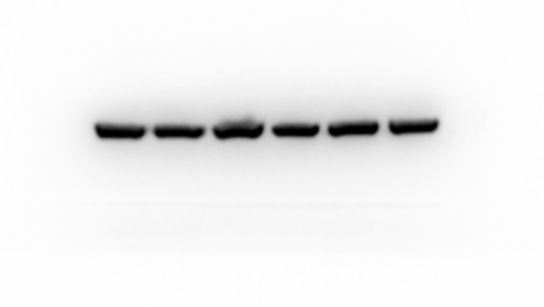


Fig. 5F GPX4(Input IP)


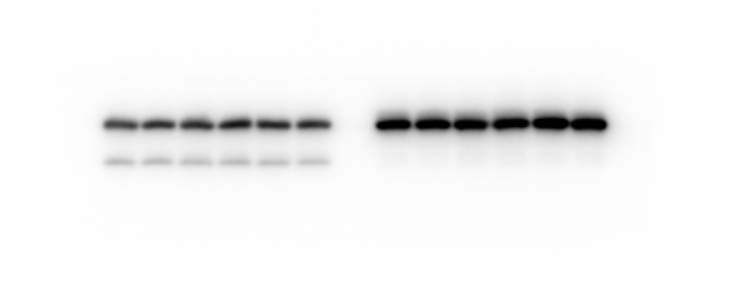


Fig. 5F MDM4(Input)


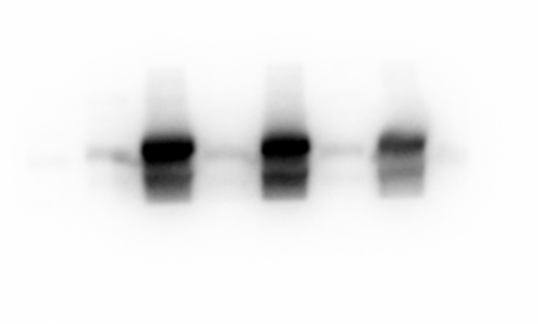


Fig. 5F Ub input


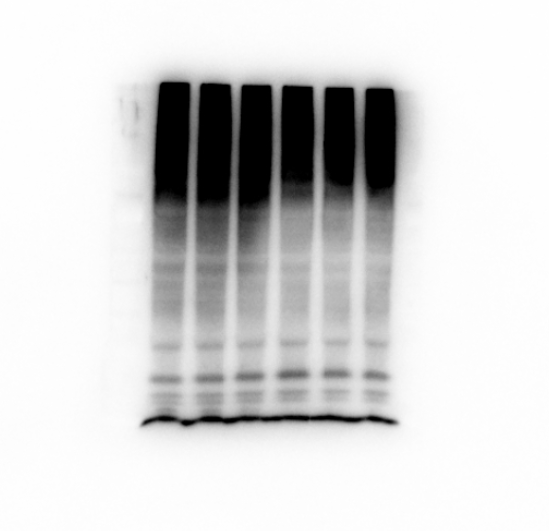


Fig. 5F


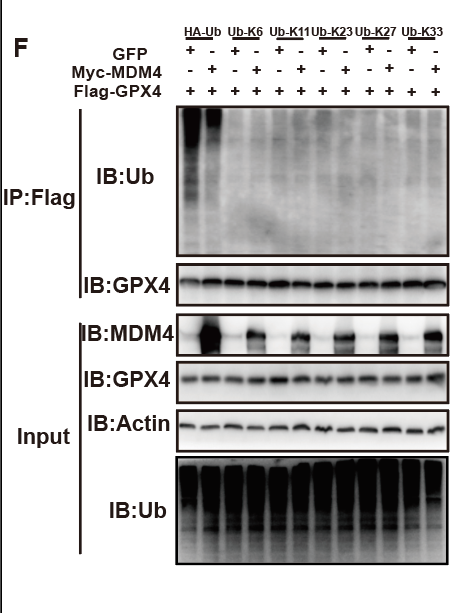


Fig. 5F Ub


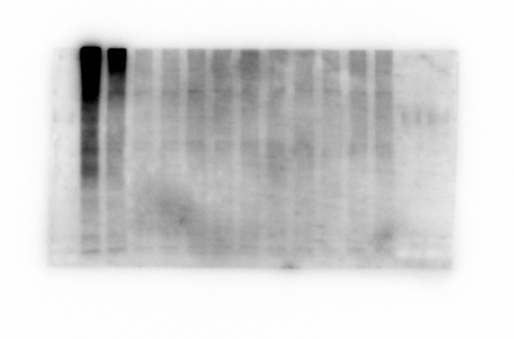


Fig. 5F Actin


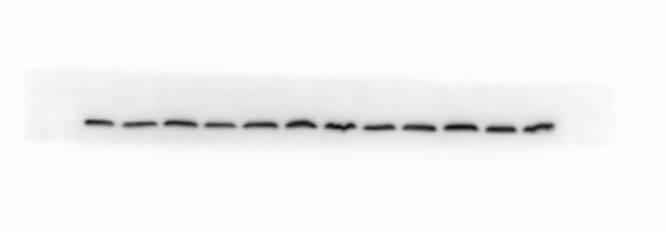


Fig. 5F GPX4(Input)


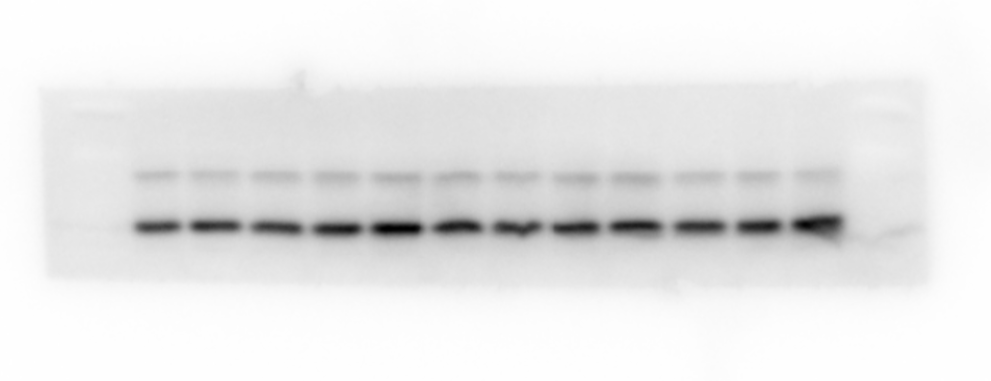


Fig. 5F GPX4(IP)


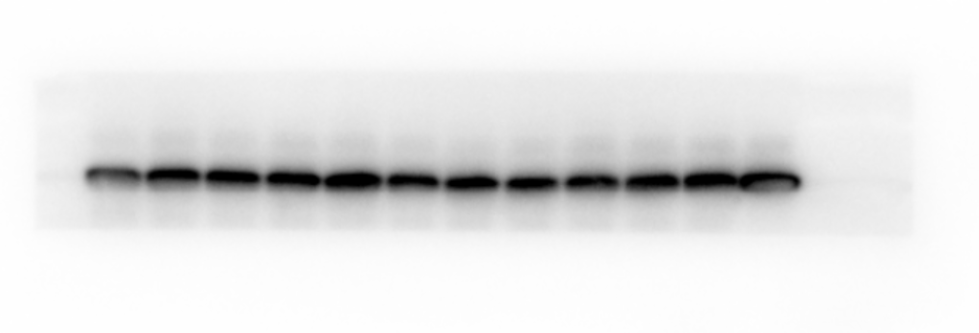


Fig. 5F MDM4(Input)


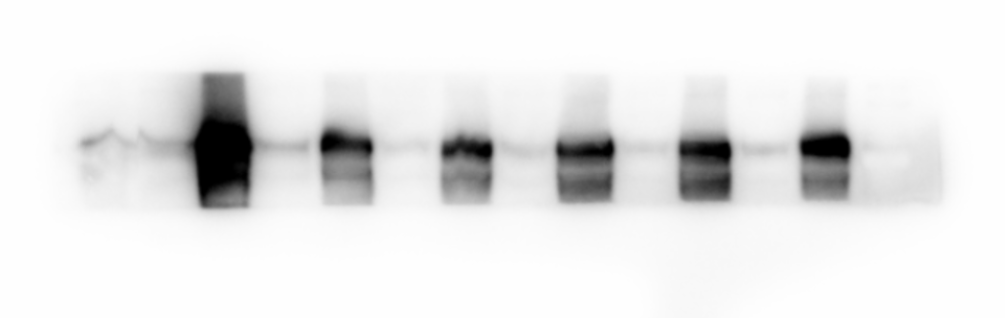


Fig. 5F Ub input


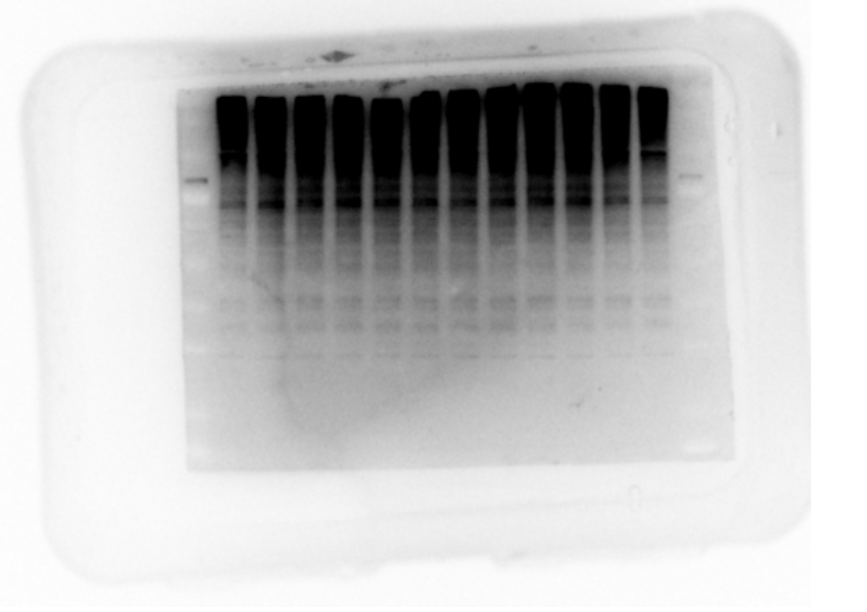


Fig. 5G


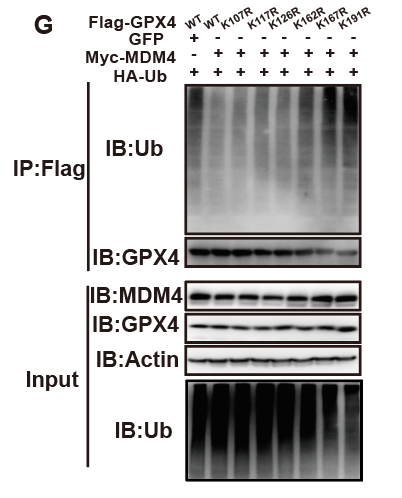


Fig. 5G IP:UB


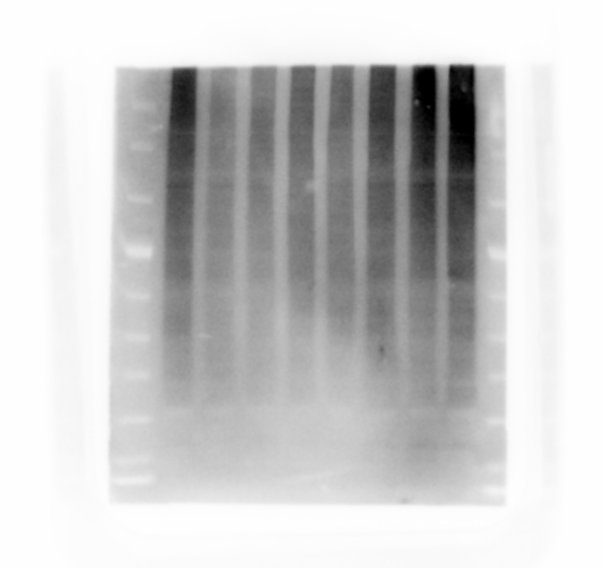


Fig. 5G Actin


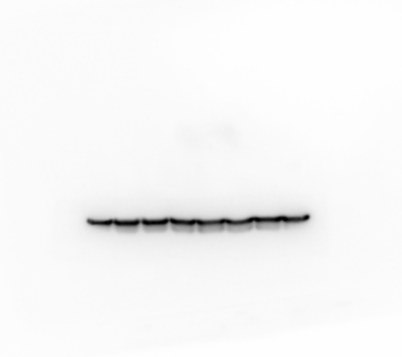


Fig. 5G GPX4 Input


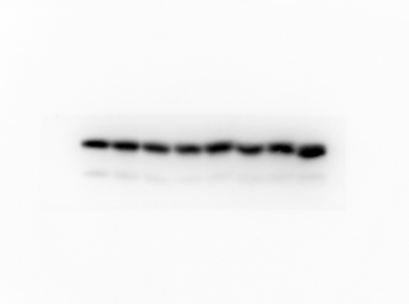


Fig. 5G GPX4 IP


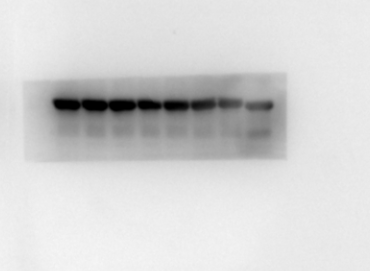


Fig. 5G MDM4 Input


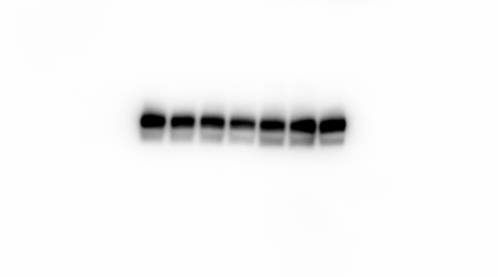


Fig. 5G Ub input


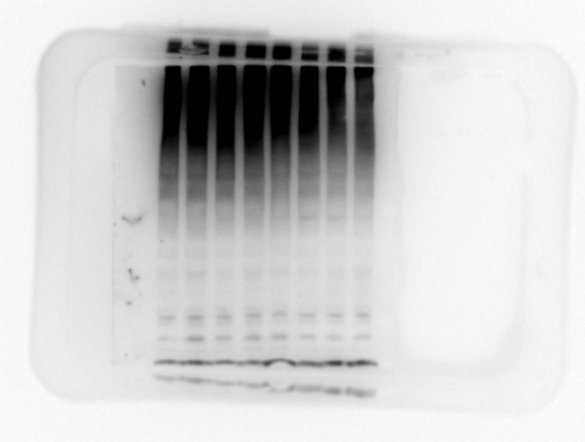


Fig. 6A


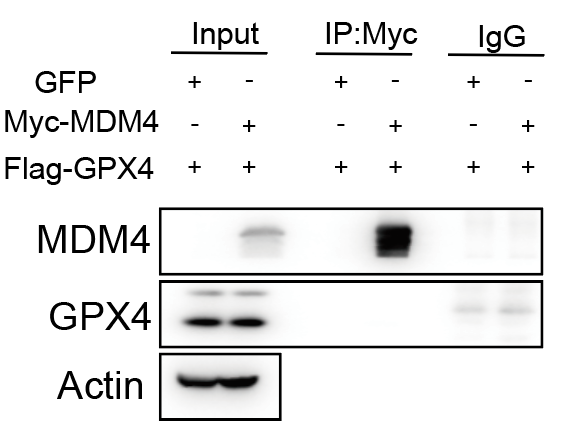


Fig. 6A Actin


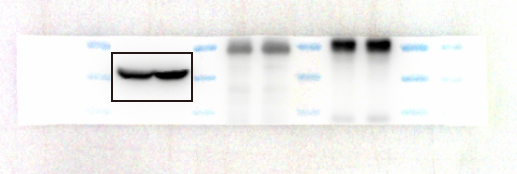


Fig. 6A GPX4


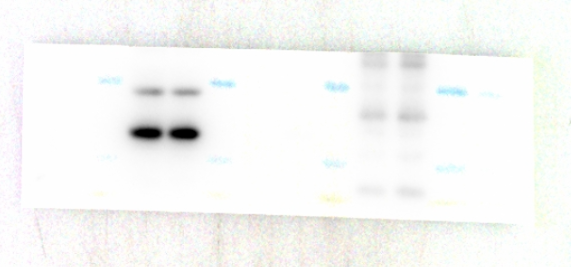


Fig. 6A MDM4


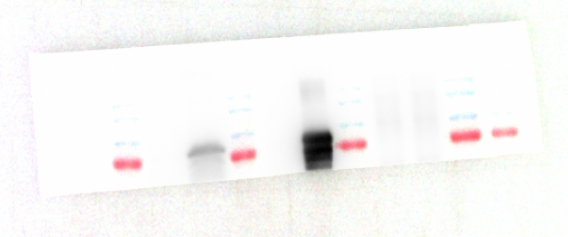


Fig. 6B


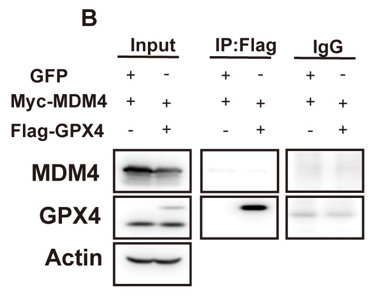


Fig. 6B Actin


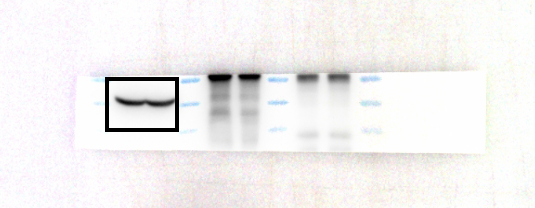


Fig. 6B GPX4


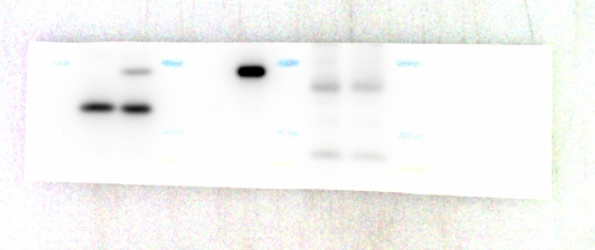


Fig. 6B MDM4


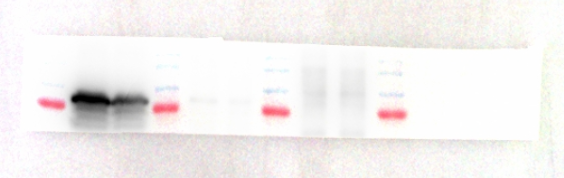


Fig. 6C


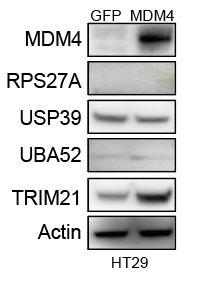


Fig. 6C Actin


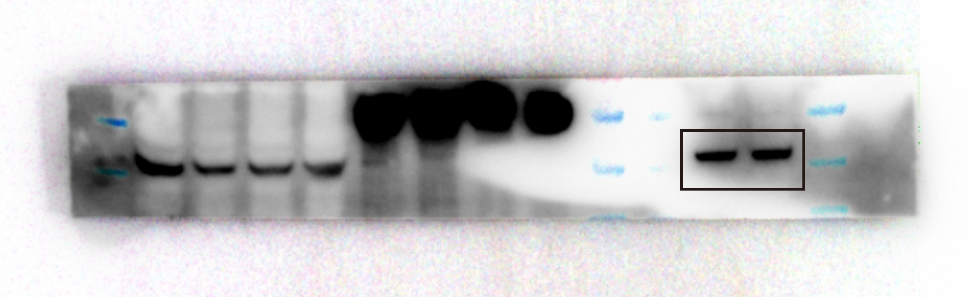


Fig. 6C TRIM21


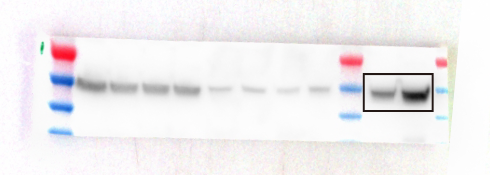


Fig. 6C UBA52


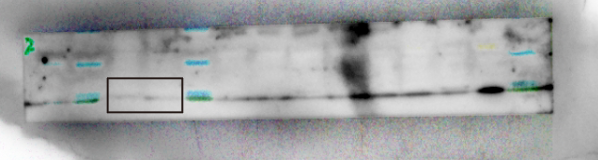


Fig. 6C RPS27A


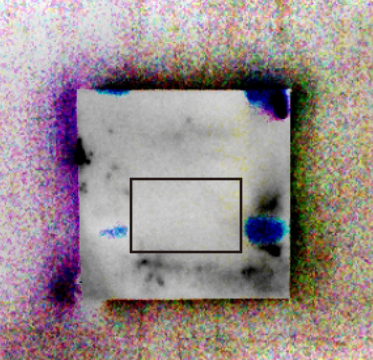


Fig. 6C USP39


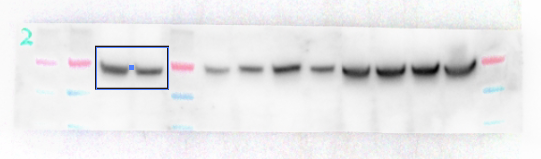


Fig. 6C MDM4


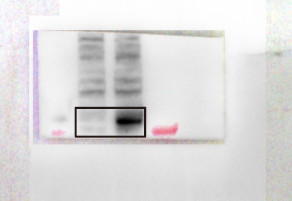


Fig. 6D


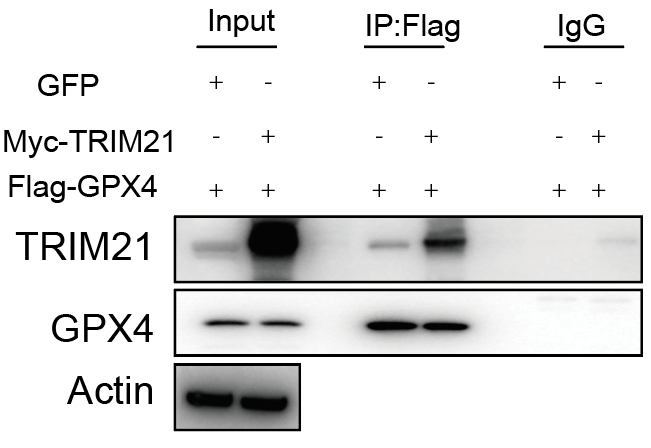


Fig. 6D TRIM21


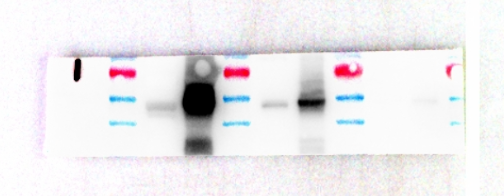


Fig. 6D GPX4


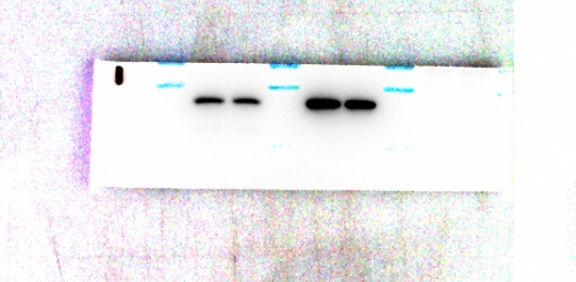


Fig. 6D Actin


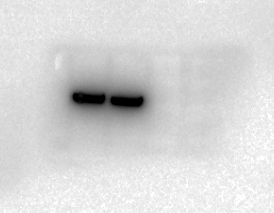


Fig. 6E


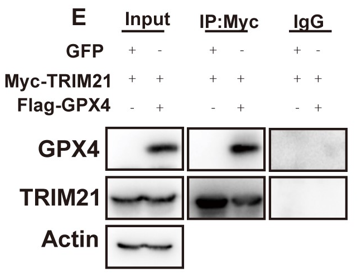


Fig. 6E Actin


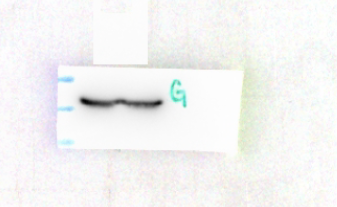

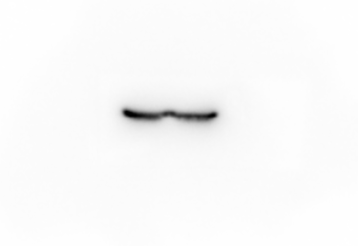


Fig. 6E GPX4(IgG)


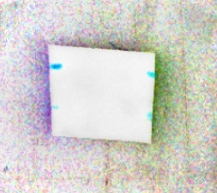

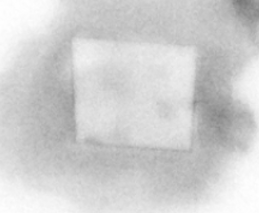


Fig. 6E GPX4(IP)


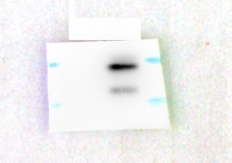


Fig. 6E GPX4(Input)


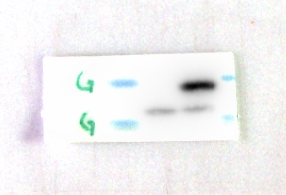


Fig. 6E TRIM21(Input)


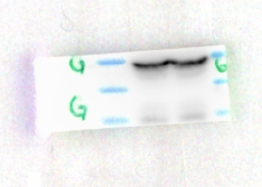


Fig. 6E TRIM21(IP)


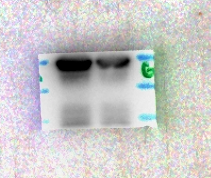


Fig. 6F


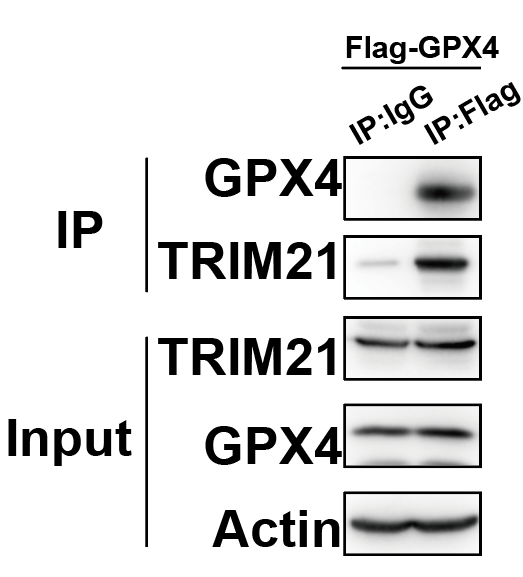


Fig. 6F Actin


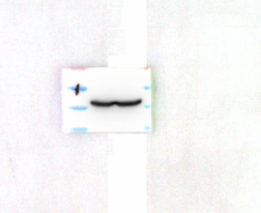


Fig. 6F Gpx4(Input)


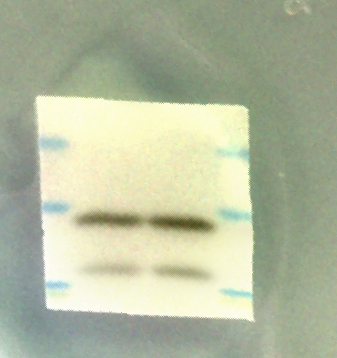


Fig. 6F GPX4(ip)


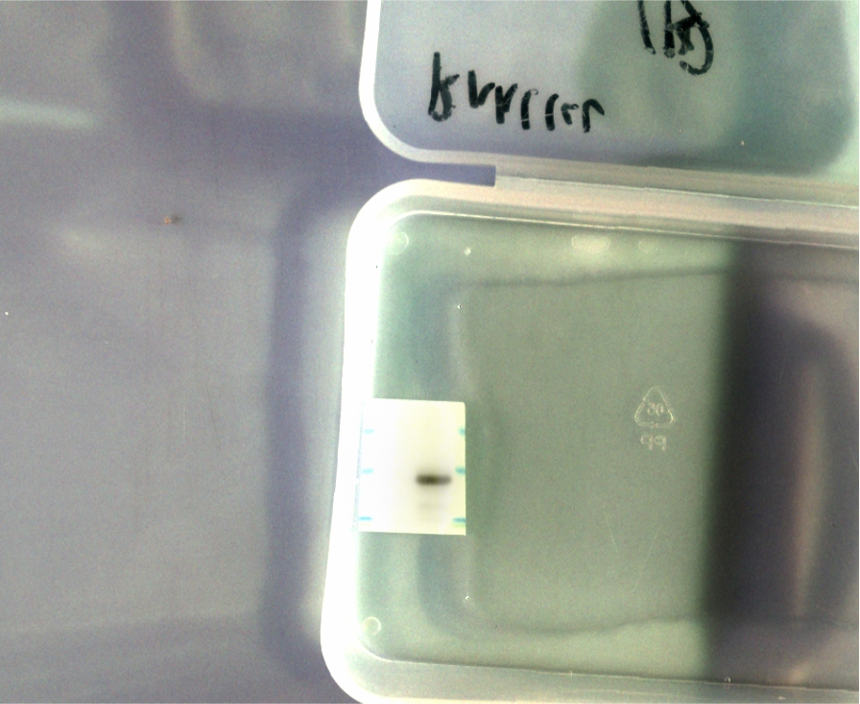


Fig. 6F TRIM21(Input)


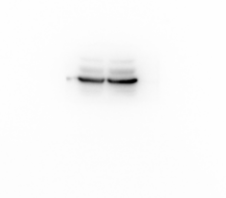


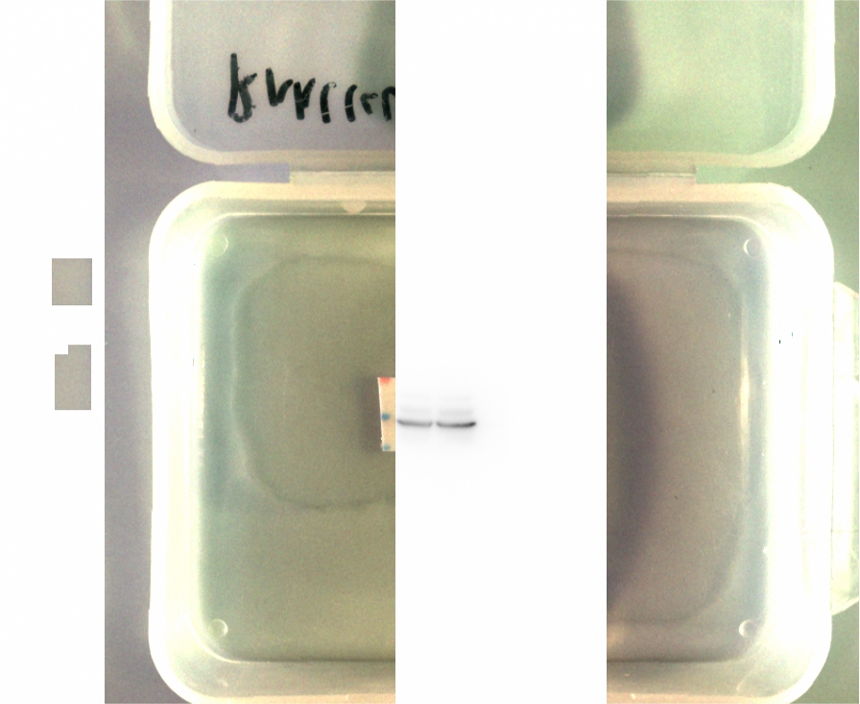


Fig. 6F TRIM21(IP)


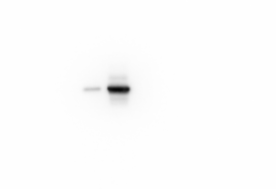


Fig. 6G


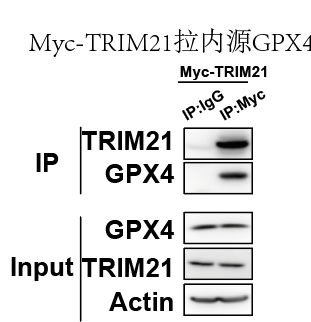


Fig. 6G Actin


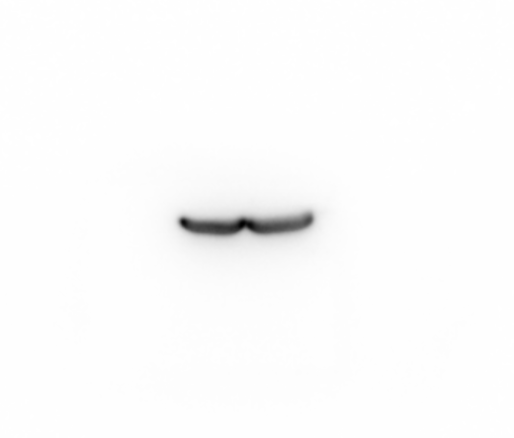


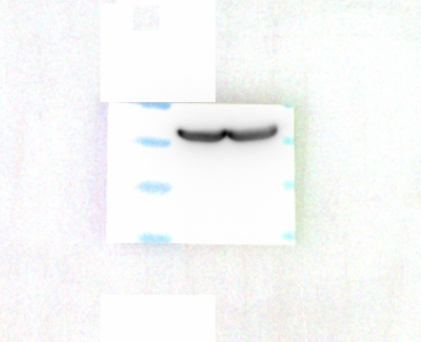


Fig. 6G GPX4(IP)


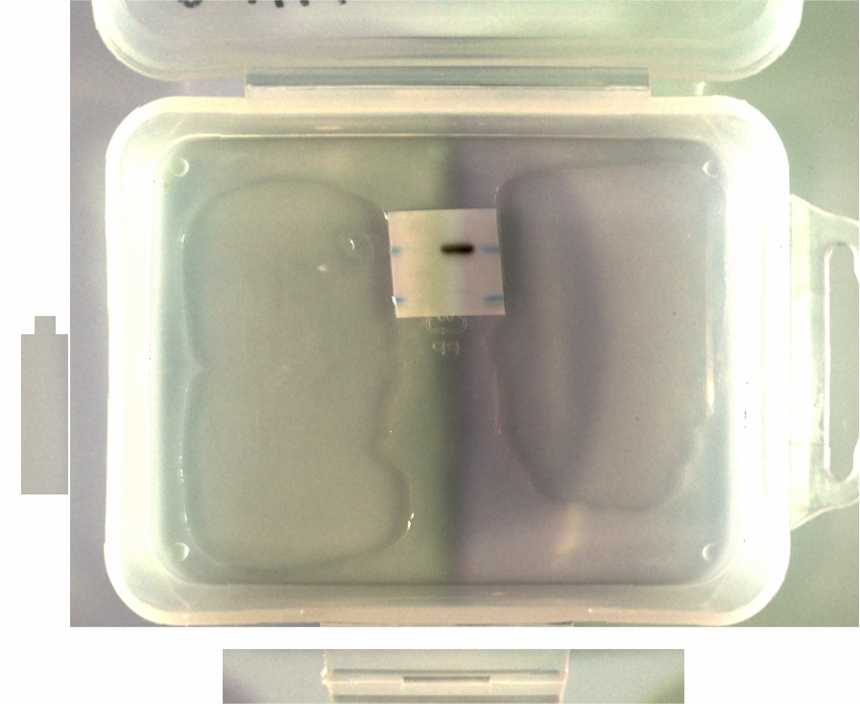


Fig. 6G TRIM21(INPUT)

Fig. 6G GPX4(INPUT)

Fig. 6G TRIM21(IP)

Fig. 6H

Fig. 6H Actin

Fig. 6H MDM4(INPUT)

Fig. 6H MDM4(IP)

Fig. 6H MDM4(IgG)

Fig. 6H TRIM21(INPUT)

Fig. 6H TRIM21(IP)

Fig. 6H TRIM21(IgG)

Fig. 6I

Fig. 6I Actin

Fig. 6I MDM4 IgG

Fig. 6I MDM4 Input

Fig. 6I TRIM21 Input

Fig. 6I TRIM21

Fig. 6I MDM4 IP

Fig. 6J

Fig. 6J Actin

Fig. 6J MDM4(IP)

Fig. 6J MDM4(Input)

Fig. 6J TRIM21(IP)

Fig. 6J TRIM21(Input)

Fig. 7B

Fig. 7B TRIM21

Fig. 7B Actin

Fig. 7B GPX4

Fig. 7C

Fig. 7C Actin

Fig. 7C GPX4

Fig. 7C TRIM21

Fig. 7D

Fig. 7D Ub

Fig. 7D Actin

Fig. 7D TRIM21(Input)

Fig. 7D GPX4(SE)

Fig. 7D GPX4(LE)

Fig. 7D Ub input

Fig. 7E

Fig. 7E UB IP

Fig. 7E Actin

Fig. 7E GPX4 Input

Fig. 7E GPX4 IP

Fig. 7E TRIM21 Input

Fig. 7E Ub input

Fig. 7F

Fig. 7F Actin

Fig. 7F GPX4

Fig. 7F TRIM21

Fig. 7F MDM4

Fig. 8A

Fig. 8A GPX4

Fig. 8A Actin

Fig. S1A

Fig. S1A MDM4

Fig. S1A Actin

Fig. S1A

Fig. S1A Actin

Fig. S1A MDM4

Fig. S4A

Fig. S4A GPX4

Fig. S4A MDM4

Fig. S4A Actin

Fig. S6A

Fig. S6A Actin

Fig. S6A MDM4

Fig. S6A TRIM21
