# Supplementary material for: MDM4 inhibits ferroptosis in p53 mutant colon cancer via regulating TRIM21/GPX4 expression
Source: Cell Death Dis. 2024 Nov 14;15(11):825. doi: 10.1038/s41419-024-07227-y (PMC11564821; doi:10.1038/s41419-024-07227-y)
Supplement: Supplementary file 2 — Supplementary material [file 41419_2024_7227_MOESM2_ESM.pdf]

Supplementary Material

Supplementary Table 1. Primer sequences.

GAPDH	human	forward	5'- GATTCCACCCATGGCAAATTC-3'
		reverse	5'- CTGGAAGATGGTGATGGGATT-3'
MDM4	human	forward	5'- GCCTTGAGGAAGGATTGGTA-3'
		reverse	5'- TCGACAATCAGGGACATCAT-3'
GPX4	human	forward	5'- TGGGAAATGCCATCAAGTGG -3'
		reverse	5'- GGTCCTTCTCTATCACCAGGGG -3'

Supplementary Table 2. Relevant antibodies.

Antibody	Company and Country	Cat NO.	Dilution Ratio
MDM4	Proteintech, China	17914-1-AP	1:1000
MDM4	Bethyl Laboratories, Germany	A300-287A	1:5000
GPX4	Proteintech, China	67763-1-Ig	1:1000
GPX4	CST, America	52455S	1:1000
TRIM21	Proteintech, China	12108-1-AP	1:1000
Ub	Proteintech, China	10201-2-AP	1:800
β-Actin	Proteintech, China	23660-1-AP	1:5000

Supplementary Table 3. Lists of shRNA Target Sequence for MDM4 and TRIM21.

sh-MDM4-1	AAGAACTACAGAAGACGATAT
sh-MDM4-2	AAAGATTCAGCTGGTTATTAA
sh-TRIM21-1	GTGGCATGGTCTCCTTCT
sh-TRIM21-2	GAAGAGAGATTTGATAGTTAT

Supplementary Figure S1

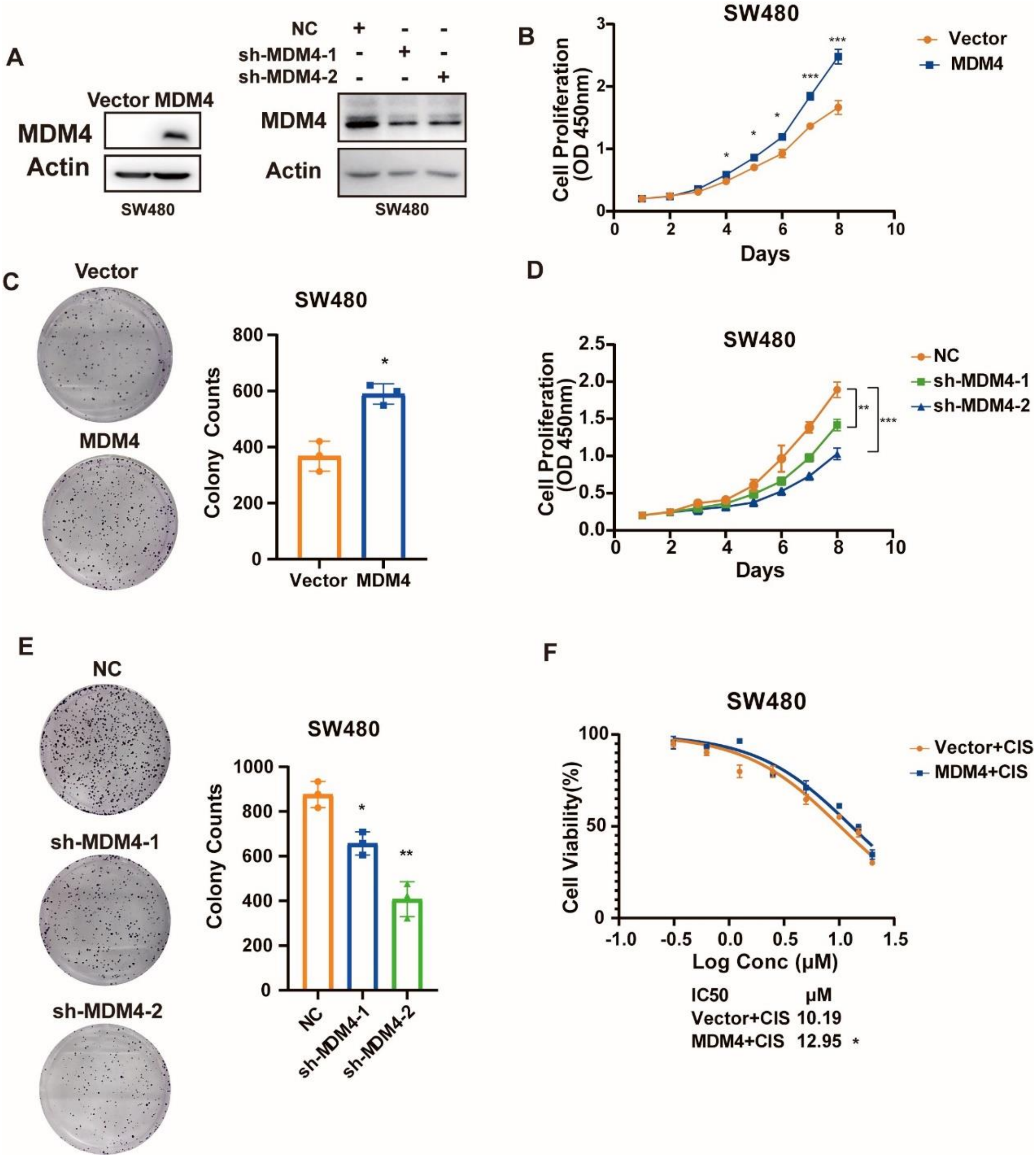

Supplementary Figure S1. MDM4 promotes growth of colon cancer cells harboring mutant p53. (A)Western blot showing MDM4 expression in stable transfected cell lines. (B) Cell viability assessed by CCK8 assay demonstrating the effect of MDM4 overexpression on cell growth (n=3). (C) Colony formation assay illustrating the effect of MDM4 overexpression on colon cancer cell proliferation (n=3). (D) Cell viability assessed by CCK8 assay demonstrating the effect of MDM4 knockdown on cell growth (n=3). (E) Colony formation assay illustrating the effect of MDM4 knockdown on colon cancer cell proliferation (n=3). (F) Overexpression of MDM4 enhances resistance to cisplatin in SW480 cells (n=3). ns: P＞0.05; *: P<0.05; **: P<0.01; ***: P<0.001. (B, C and F) T test (E) One-way ANOVA were used for statistical analysis.

Supplementary Figure S2

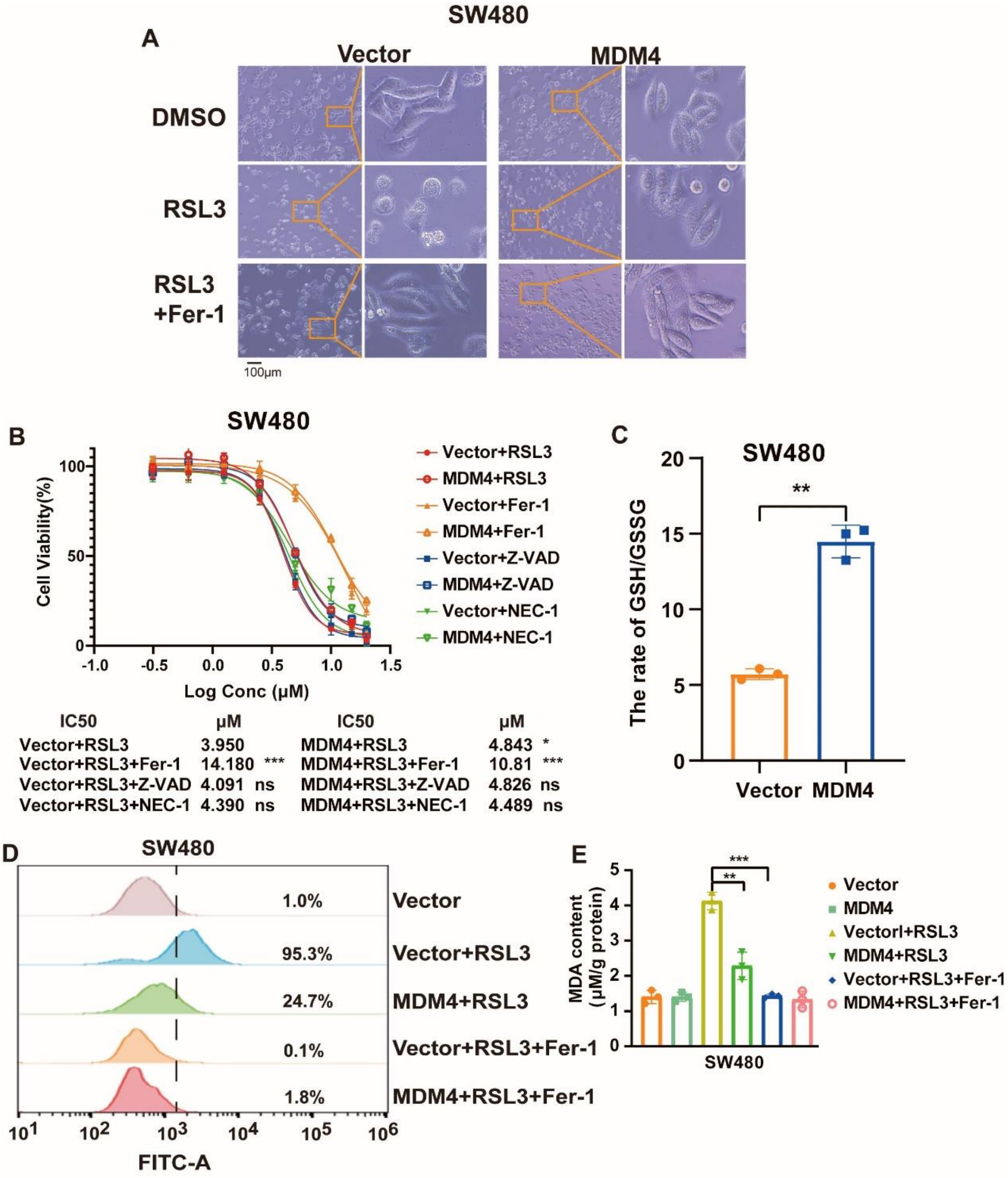

Supplementary Figure S2. Overexpression of MDM4 inhibits ferroptosis in p53 mutant colon cancer cells. (A) Microscopic images demonstrating cell morphology changes in SW480 cells after RSL3 treatment with or without MDM4 overexpression. (B) Cell viability of SW480 cells treated with different doses of RSL3 alone, or in combination with Fer-1 or Z-VAD for 48 hours (n=3). (C) Effect of MDM4 overexpression on GSH/GSSG ratio in SW480 cells (n=3). (D) Flow cytometry analysis of ROS production in MDM4 overexpression cells. (E) MDA content measured in MDM4 overexpression cells (n=3). ns: $P>0.05$; *: $P<0.05$; **: $P<0.01$; ***: $P<0.001$. (B, E) One-way ANOVA and (C) T test were used for statistical analysis.

Supplementary Figure S3

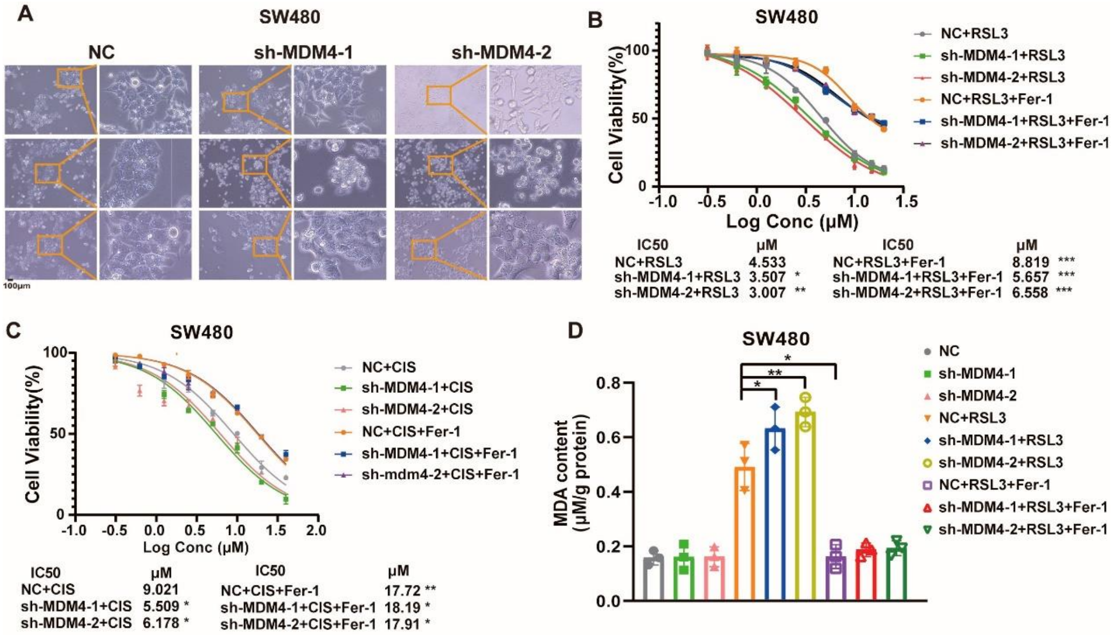

Supplementary Figure S3. Knockdown MDM4 increased ferroptosis sensitivity of p53 mutant colon cancer cells. (A) Microscopic images showing cell morphology changes in SW480 cells after RSL3 treatment with or without MDM4 knockdown. (B) Cell viability of SW480 cells treated with different doses of RSL3 alone, or in combination with Fer-1 for 48 hours after MDM4 knockdown (n=3). (C) Cell viability of SW480 cells treated with different doses of cisplatin (CIS) for 48 hours after MDM4 knockdown (n=3). (D) MDA content measured in MDM4 knockdown cells (n=3). ns: *P*＞0.05; *: *P*<0.05; **: *P*<0.01; ***: *P*<0.001. (B, C and D) One-way ANOVA were used for statistical analysis.

Supplementary Figure S4

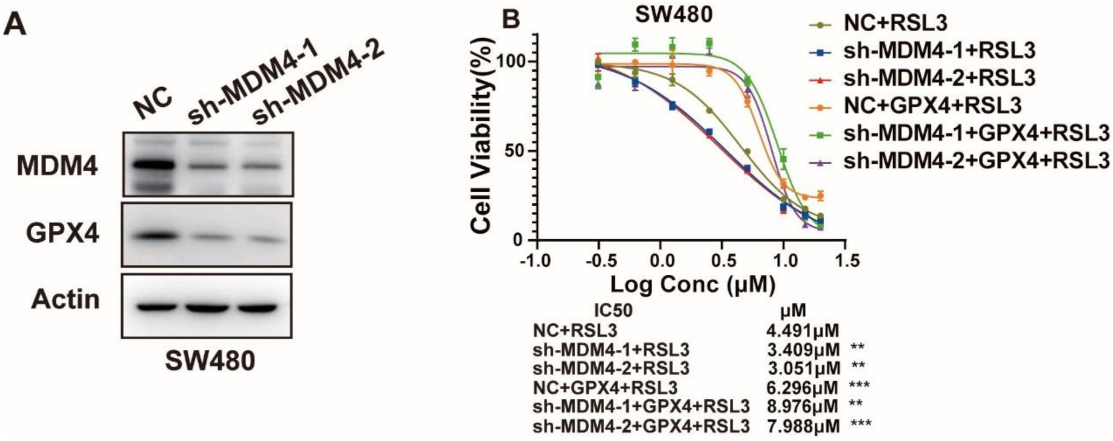

Supplementary Figure S4. MDM4 inhibits ferroptosis by upregulating GPX4 protein expression level. (A) Western blot showed the effect of MDM4 knockdown on GPX4 protein expression levels in SW480 cells. (B) Effects of GPX4 overexpression on RSL3 resistance in colon cancer cells following MDM4 knockdown (n=3). ns: $P > 0.05$; *: $P<0.05$; **: $P<0.01$; ***: $P<0.001$. (B) One-way ANOVA was used for statistical analysis.

Supplementary Figure S5

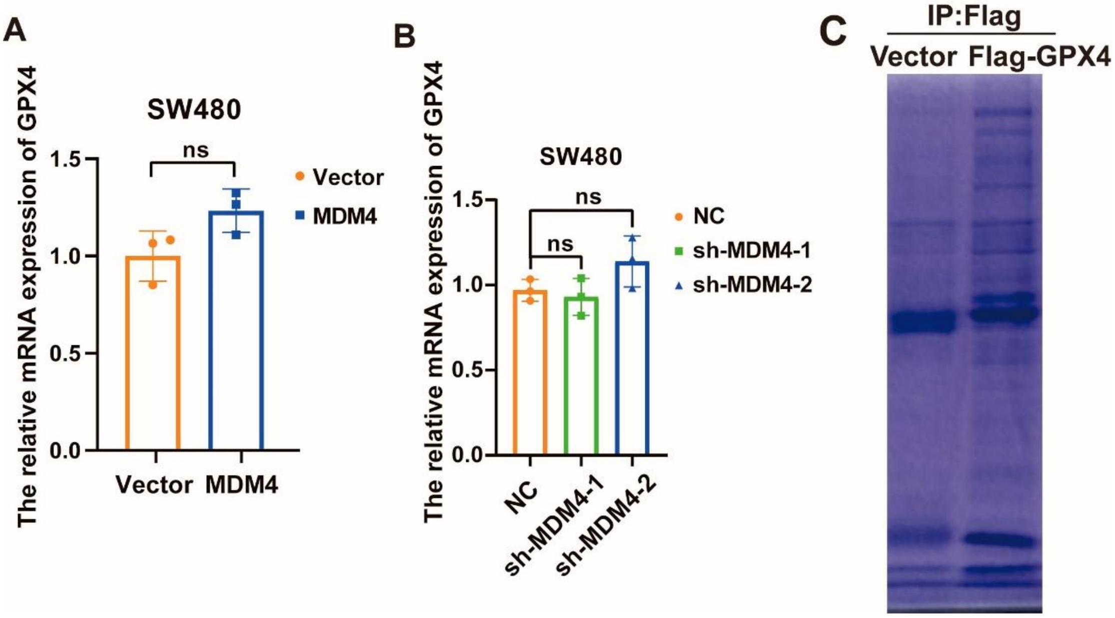

Supplementary Figure S5. MDM4 has no effect on GPX4 mRNA level. (A) Effect of MDM4 overexpression on GPX4 mRNA levels in SW480 cells (n=3). (B) Effect of MDM4 knockdown on GPX4 mRNA levels in SW480 cells (n=3). (C) Coomassie blue staining of proteins immunoprecipitated with Flag-GPX4 followed by mass spectrometry analysis. ns: $P>0.05$; *: $P<0.05$; **: $P<0.01$; ***: $P<0.001$. (A) T test and (B) One-way ANOVA were used for statistical analysis.

Supplementary Figure S6

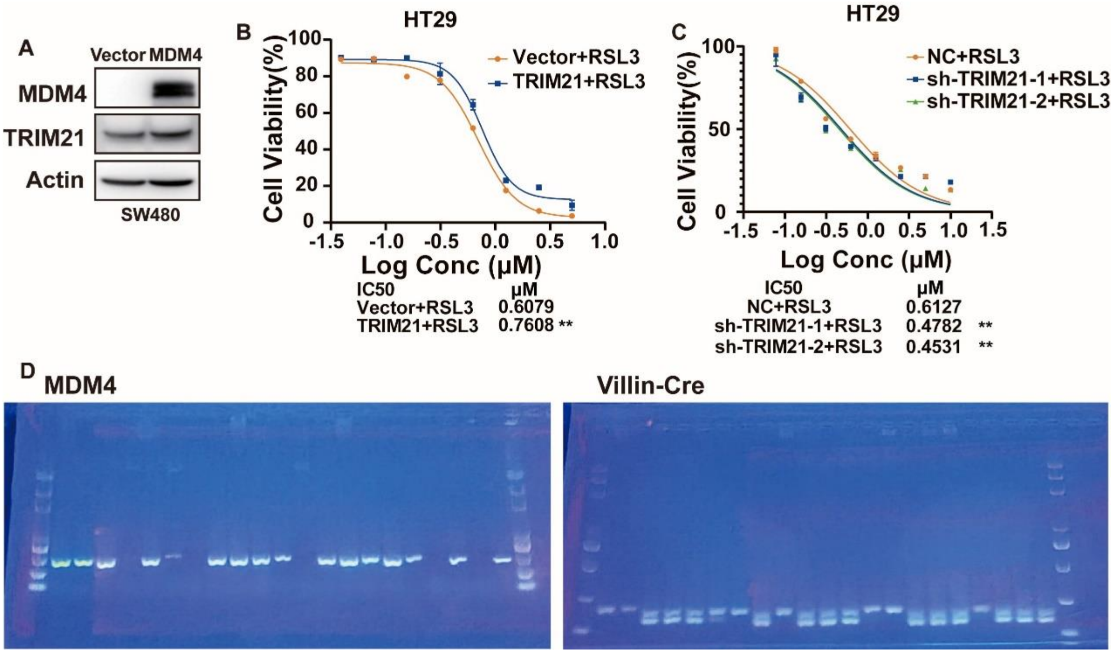

Supplementary Figure S6. MDM4 upregulates TRIM21 expression, effect of TRIM21 on ferroptosis and mouse genotyping. (A) Effect of MDM4 overexpression on the expression level of TRIM21 in SW480 cells. (B) Cell viability of SW480 cells overexpressing TRIM21 treated with different doses of RSL3 for 48 hours (n=3). (C) Cell viability of SW480 cells with TRIM21 knockdown treated with different doses of RSL3 for 48 hours (n=3). (D) Genotyping of tissue-specific expression mice. ns: *P*＞0.05; *: *P*<0.05; **: *P*<0.01; ***: *P*<0.001. (B) T test and (C) One-way ANOVA were used for statistical analysis.
